# Synthesis, Characterization and Antimicrobial and Anticancer Evaluations of Some Novel Heteroannulated Difuro[3,2-*c*:3′,2′-*g*]Chromenes

**DOI:** 10.3390/molecules29102319

**Published:** 2024-05-15

**Authors:** Najla A. Alshaye, Magdy A. Ibrahim, Al-Shimaa Badran

**Affiliations:** 1 Department of Chemistry, College of Science, Princess Nourah bint Abdulrahman University, P.O. Box 84428, Riyadh 11671, Saudi Arabia; naalshaye@pnu.edu.sa; 2 Department of Chemistry, Faculty of Education, Ain Shams University, Roxy, Cairo 11711, Egypt; elshimaabadran@edu.asu.edu.eg

**Keywords:** Friedländer, active methylene ketones, cyclocondensation furochromenofuropyridines, furochromenofuropyridopyrimidines

## Abstract

The goal of this study was directed to synthesize a novel class of annulated compounds containing difuro[3,2-*c*:3′,2′-*g*]chromene. Friedländer condensation of *o*-aminoacetyl derivative **3** was performed with some active methylene ketones, namely, 1,3-cyclohexanediones, pyrazolones, 1,3-thiazolidinones and barbituric acids, furnished furochromenofuroquinolines (**4**,**5**), furochromenofuropyrazolopyridines (**6**–**8**), furochromenofurothiazolopyridines (**9**,**10**) and furochromenofuropyridopyrimidines (**11**, **12**), respectively. Also, condensation of substrate **3** with 5-amine-3-methyl-1*H*-pyrazole and 6-amino-1,3-dimethyluracil, as cyclic enamines, resulted in polyfused systems **13** and **14**, respectively. In vitro antimicrobial efficiency of the prepared heterocycles against microbial strains exhibited variable inhibition action, where compound **3** was the most effective against all kinds of microorganisms. A significant cytotoxic activity was seen upon the annulation of the starting compound with thiazolopyridine (**9** and **10**) as well as pyridopyrimidine moieties (**11**, **12** and **14**). The spectroscopic and analytical results were used to infer the structures of the novel synthesized compounds.

## 1. Introduction

**Figure 1:**
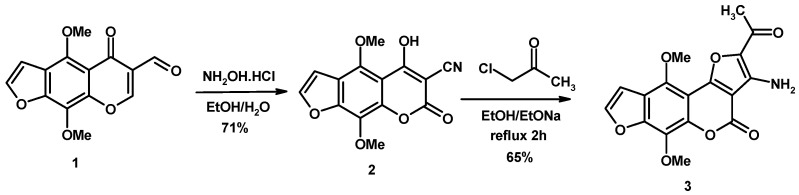
Synthetic pathway for the starting substrate **3**.

Visnagin and khellin are furochromenes, named as 4-methoxy/4,9-dimethoxy-7-methyl-5*H*-furo[3,2-*g*]chromen-5-ones. The most important natural source of visnagin and khellin is *Ammi visnaga* seeds [1,2] that are employed as pharmaceuticals to treat kidney and bladder stones as well as diuretic infusions [3,4]. Khellin has amazing benefits on the urinary system’s smooth muscles and slows down the formation of calcium oxalate crystals, which are the vital cause of common kidney stones [5]. Significant antispasmodic and coronary vasodilator effects have been observed for khellin [6,7]. Furochromones have been extensively studied as antimicrobial [8], anticancer [9], anti-inflammatory [10], antiviral [11] and anticonvulsant agents [12]. Additionally, a range of investigations have been conducted on the photodynamic characteristics of khellin [13]. Khellin, a photochemotherapeutic, is frequently used to treat vitiligo and psoriasis, as well as skin diseases [14,15]. Furo[3,2-*g*]chromenes have been examined for their solvatochromic, electronic and photoelectrical characteristics, as well as theoretical DFT calculations [16,17]. A wide range of furo[3,2-*g*]chromenes and benzofurans have been synthesized using khellin containing electron-withdrawing substituents at position 6 [18,19]. Compounds bearing neighboring amino and carbonyl functions widely serve as good precursors to react with active methylene ketones through Friedländer reactions [20,21,22]. In our previous works, reaction of 4,9-dimethoxy-5-oxo-5*H*-furo[3,2-*g*]chromene-6-carboxaldehyde (**1**) with hydroxylamine hydrochloride in aqueous ethanol yielded carbonitrile derivative **2** which, upon treatment with chloroacetone, yielded the target precursor **3**, as shown in Figure 1 [23,24]. The present research aimed to employ compound **3** as a precursor to form a novel category of polyfused systems through reactions with a diversity of active methylene nucleophiles as well as evaluate their antimicrobial and anticancer efficiency.

## 2. Results and Discussion

### 2.1. Characterization of the Synthesized Compounds

**Scheme 1:**
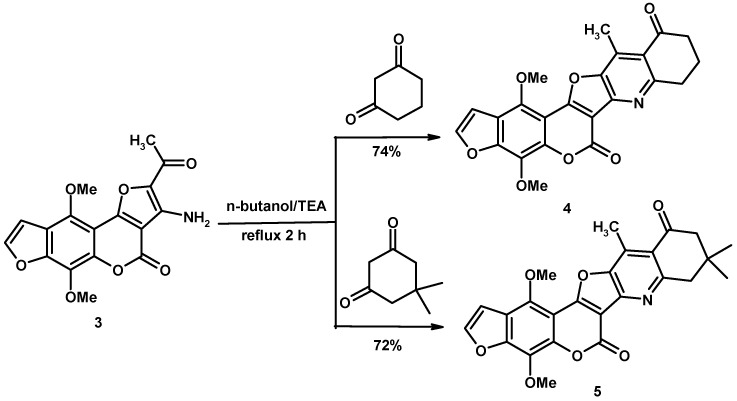
Formation of furochromenofuroquinolines **4** and **5**.

The current study aimed to investigate the chemical reactivity of compound **3** towards some cyclic methylene ketones to create a novel series of heteroannulated compounds including difuro[3,2-*c*:3′,2′-*g*]chromenes. Therefore, a condensation reaction of substrate **3** with 1,3-cyclohexanedione and 5,5-dimethyl-1,3-cyclohexanedione in boiling *n*-butanol, including triethylamine (TEA), furnished furochromenofuroquinolines **4** and **5**, respectively (Scheme 1). The IR spectra of compounds **4** and **5** exhibited characteristic absorption bands at $\tilde{v}$ 1716/1712 (C=O_α-pyrone_) and 1678/1681 cm^−1^ (C=O_quinolinone_). Compounds **4** and **5** were deduced from their mass spectra that displayed the parent ion peaks at *m*/*z* 419 and 447 that closely matched with the proposed molecular formulae C_23_H_17_NO_7_ (419.38) and C_25_H_21_NO_7_ (447.44), respectively. Their ^1^H NMR spectra showed characteristic singlet signals at δ 2.24 and 2.31, respectively, that were attributed to the CH_3pyridine_. In addition, three triplet signals due to three CH_2_ groups were seen in the spectrum of compound **4** at δ 2.02, 2.72 and 3.12. Meanwhile, three distinctive singlet signals were observed in the ^1^H NMR spectrum of compound **5** at δ 1.03 (2CH_3_), 2.74 (CH_2_) and 2.92 (CH_2_). ^13^C-NMR spectrum of compound **4** showed characteristic signals at δ 17.3 (CH_3_), 22.0 (CH_2_), 32.2 (CH_2_), 36.4 (CH_2_), 58.5 (OCH_3_), 59.4 (OCH_3_), 164.3 (OC=O) and 192.6 (C=O_quinoline_). Meanwhile, specific signals were seen in the ^13^C-NMR spectrum of compound **5** at δ 12.9 (2CH_3_), 15.3 (CMe_2_), 17.8 (CH_3_), 31.9 (CH_2_), 36.7 (CH_2_), 59.8 (OCH_3_), 60.2 (OCH_3_), 164.5 (OC=O) and 191.8 (C=O_quinolinone_).

**Scheme 2:**
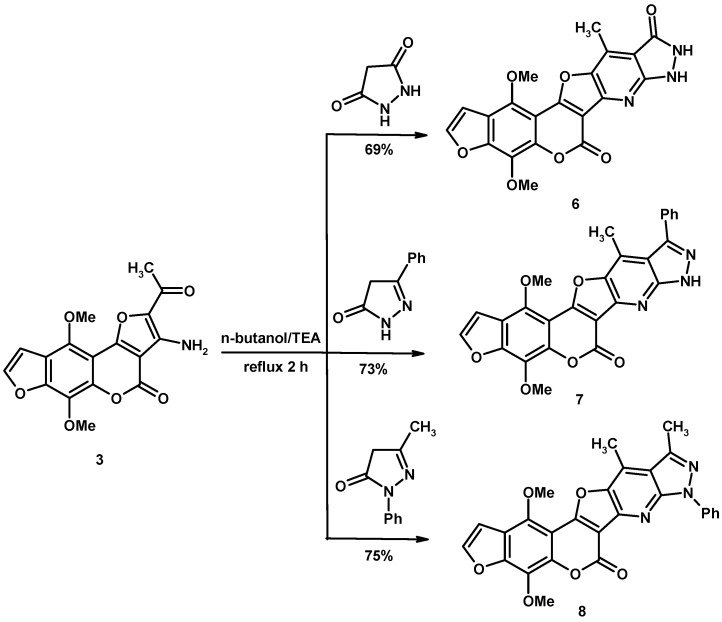
Formation of annulated furochromenofuropyrazolopyridines **6**–**8**.

Likewise, pyrazolones involving active methylene groups may act as convenient reagents to condense with precursor **3**. Thus, boiling compound **3** with some pyrazolones in refluxing *n*-butanol/TEA yielded annulated furochromenofuropyrazolopyridines **6**–**8** (Scheme 2) [25]. Their mass spectra provided a strong support to the suggested structures that displayed the molecular ion peaks, as the base peak, at *m*/*z* 407, 467 and 481, verifying their molecular formulae C_20_H_13_N_3_O_7_, C_26_H_17_N_3_O_6_ and C_27_H_19_N_3_O_6_, respectively. The IR spectra of compounds **6**–**8** displayed typical absorption bands due to C=O_α-pyrone_ at $\tilde{v}$ 1723, 1726 and 1718 cm^−1^; the spectrum of compound **6** showed definite absorption bands due to C=O_pyrazolone_ at $\tilde{v}$ 1679 cm^−1^. The ^1^H NMR spectra of products **6**–**8** presented certain singlets due to CH_3 pyridine_ at δ 2.27, 2.29 and 2.25, respectively; the spectrum of compound **8** showed an additional singlet due to CH_3 pyrazole_ at δ 2.03. The ^13^C-NMR spectrum of compound **6** showed characteristic signals at δ 17.9 (CH_3_), 59.7 (OCH_3_), 60.6 (OCH_3_), 163.3 (OC=O) and 170.6 (C=O_pyrazolone_).

**Scheme 3:**
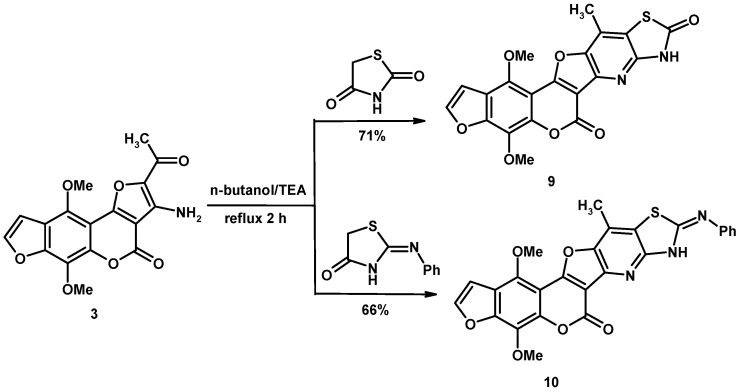
Formation of furochromenofurothiazolopyridines **9** and **10**.

Condensation of substrate **3** with 1,3-thiazolidine-2,4-dione and 2-(phenylimino)-1,3-thiazolidin-4-one, in *n*-butanol containing TEA, afforded the novel annulated furochromenofurothiazolopyridines **9** and **10**, respectively (Scheme 3) [25]. The molecular formulae C_20_H_12_N_2_O_7_S (424.38) and C_26_H_17_N_3_O_6_S (499.49) for compounds **9** and **10** were inferred from the mass spectra which presented the parent ion peaks at *m*/*z* 424 and 499, respectively. The IR spectra displayed typical absorption bands at $\tilde{v}$ 3239/3215 (NH) and 1725/1720 cm^−1^ (C=O_α-pyrone_); the spectrum of product **9** showed a definite absorption band due to C=O_thiazole_ at $\tilde{v}$ 1673 cm^−1^. The ^1^H NMR spectra of compounds **9** and **10** exhibited singlet signals at δ 2.30/2.22 (CH_3_), 3.91/3.89 (OCH_3_), 4.03/3.98 (OCH_3_) and 11.19/10.31 (NH vanished with D_2_O). The ^13^C NMR spectrum of product **10** exhibited specific signals at δ 17.8 (CH_3_), 59.3 (OCH_3_), 60.2 (OCH_3_), 164.7 (OC=O) and 171.3 (C=O_thiazole_).

**Scheme 4:**
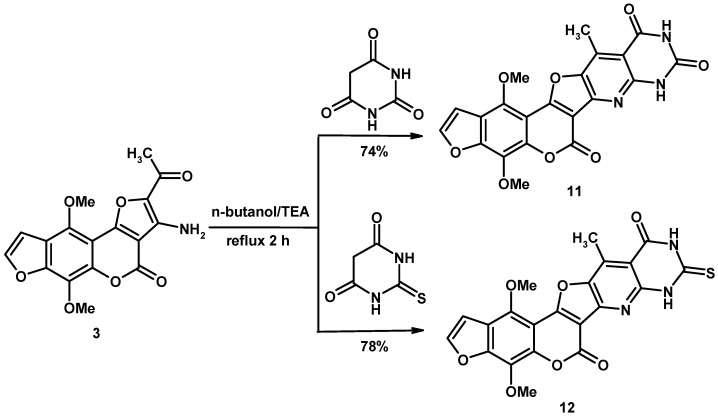
Formation of polyfused heterocycles **11** and **12**.

After that, reaction of precursor **3** with barbituric acid and thiobarbituric acid in *n*-butanol containing TEA, under reflux, yielded the polyfused heterocycles containing pyrido[2,3-*d*]pyrimidines **11** and **12** (Scheme 4). The products **11** and **12** were established through mass spectra which revealed the parent ion peaks at *m*/*z* 435 and 451, as the base peaks, that coincide well with their assigned molecular formulae C_21_H_13_N_3_O_8_ (435.34) and C_21_H_13_N_3_O_7_S (451.41). The IR spectra of **11** and **12** revealed distinctive absorption bands at $\tilde{v}$ 1722/1716 (C=O_α-pyrone_) and 1691/1687 cm^−1^ (C=O_pyrimidine_). Their ^1^H NMR spectra displayed definite singlet signals at δ 2.31/2.27 (CH_3_), 3.91/3.92 (OCH_3_) and 3.98/4.01 (OCH_3_) as well as two definite doublets due to H-3_furan_ and H-2_furan_ at δ 7.25/7.21 and 8.03/7.96, respectively. The 2NH protons were seen as D_2_O-vanished signals at δ 10.75 and 11.03. The ^13^C NMR spectrum of compound **12** exhibited specific signals at δ 17.1 (CH_3_), 59.7 (OCH_3_), 60.3 (OCH_3_), 164.0 (OC=O), 171.7 (C=O_pyrimidine_) and 185.2 (C=S).

**Scheme 5:**
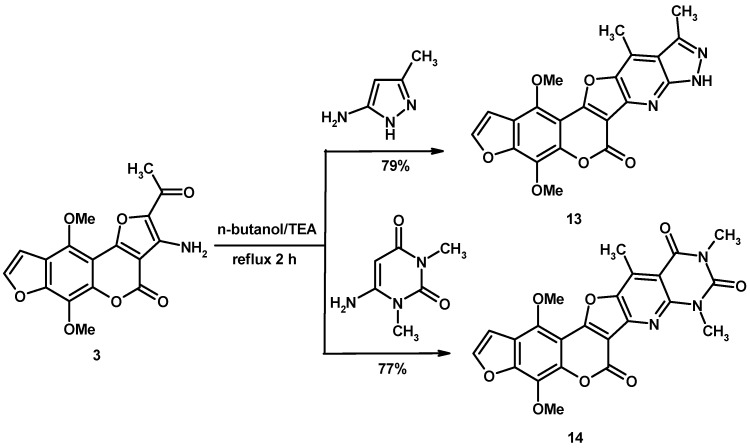
Formation of polyfused compounds **13** and **14**.

Finally, boiling precursor **3** with 5-amine-3-methyl-1*H*-pyrazole and 6-amino-1,3-dimethyluracil in *n*-butanol/TEA, under reflux, yielded heteroannulated compounds **13** and **14**, respectively (Scheme 5) [26]. The mass spectra can be considered as strong evidence for structures **13** and **14** that exhibited the molecular ion peaks at *m*/*z* 405 and 463, verifying their molecular formulae C_21_H_15_N_3_O_6_ (405.36) and C_23_H_17_N_3_O_8_ (463.41), respectively. The ^1^H NMR spectrum of product **13** presented specific four singlet signals in the upfield region at δ 2.20 (CH_3_), 2.31 (CH_3_), 3.91 (OCH_3_) and 4.02 (OCH_3_), while the spectrum of product **14** displayed five singlet signals at δ 2.35 (CH_3_), 3.06 (NCH_3_), 3.19 (NCH_3_), 3.92 (OCH_3_) and 4.06 (OCH_3_). The IR spectrum of product **14** revealed definite absorption bands at $\tilde{v}$ 1723 (C=O_α-pyrone_) and 1704, 1689 (2C=O_pyrimidine_).

### 2.2. Antimicrobial Evaluation

The efficacy of the present compounds as antimicrobial agents was assessed by screening against Gram-positive bacteria like *Staphylococcus aureus* and *Bacillus subtilis*, Gram-negative bacteria like *Salmonella typhimurium* and *Escherichia coli*, yeast like *Candida albicans*, and fungus like *Asperigillus fumigatus*. It is appropriate to measure the inhibition zones along with the disc diameter (6 mm), using the disc agar diffusion method, in order to estimate the antimicrobial efficacy (Table 1) [27].

The data of antimicrobial results depicted in Table 1 showed that: the precursor **3** offered excellent efficacy against all types of the selected microbes, and this could be attributed to the presence of the difuro[3,2-*c*:3′,2′-*g*]chromene moiety bearing specific functional groups. Meanwhile, all the synthesized products did not show such activity against all microorganisms. Compounds **6**–**8** and **13** presented significant efficacy against Gram-negative bacteria, fungus and yeast; this could be attributed to the presence of furochromenofuropyrazolopyridines with a high percent of nitrogen content. Moreover, compounds **9** and **10** offered high activity against Gram-negative bacteria, which might be attributable to the presence of furochromenofurothiazolopyridine moieties. Further, compounds **11**, **12** and **14** displayed high activity against Gram-positive bacteria, which might be attributable to the presence of furochromenofuropyridopyrimidine moieties. Consequently, certain molecules that were synthesized may find application as antibacterial agents.

### 2.3. Anticancer Evaluation

The in vitro anticancer activity of the synthesized polyfused heterocyclic compounds including difuro[3,2-*c*:3′,2′-*g*]chromenes was evaluated against colon cancer HCT-116 and hepatocellular carcinoma (liver) HepG2 cell lines. The cytotoxic activity was evaluated using MTT colorimetric assay [28]. The data obtained for the cytotoxic activities (IC_50_ values) were compared with the reference anticancer medication (Doxorubicin). The results shown in Table 2 demonstrated that: the starting precursor, difuro[3,2-*c*:3′,2′-*g*]chromene **3**, presents moderate cytotoxic efficiency against the two selected cell lines (HCT-116 and HepG2). Annulation of compound **3** with the other heterocyclic ring altered the cytotoxicity against the cancer cell lines. Compounds **4** and **5** displayed good anticancer efficacy and this might be attributed to the annulation of the starting compound with quinoline moieties. Also, compounds **6**–**8** and **13** presented good activity towards the selected cancer cell lines and this might be assigned to the annulation of compound **3** with pyrazolopyridine moieties.

On the other hand, compounds **9** and **10** showed excellent cytotoxic activity, and this might be assigned to the annulation of compound **3** with thiazolopyridine moieties. Also, excellent efficiency was recorded as presented in compounds **11**, **12** and **14** and this might be attributed to the annulation of the staring compound with pyridopyrimidine moieties.

As aforementioned above, remarkable inhibitory effect was observed when the staring compound **3** is annulated with heterocyclic systems, especially thiazolopyridines as well as pyridopyrimidines.

## 3. Materials and Methods

### 3.1. General Information

General. A digital Stuart SMP3 device (Büchi, Flawil, Switzerland) was employed to determine the melting points of the synthesized compounds. Using KBr discs, the infrared spectra were obtained using an FTIR Nicolet IS10 spectrophotometer (cm^−1^) (Thermo Fisher Scientific, Waltham, MA, USA). The Mercury-400BB apparatus (vnmr1, Rheinstetten, Germany) was used to measure the ^1^H NMR (400 MHz) and ^13^C NMR (100 MHz) spectra. The solvent used was DMSO-*d*_6_, and the internal standard was TMS (δ, ppm). The GC-2010 Shimadzu Gas Chromatography Instrument Mass Spectrometer (70 eV) (Manchester, UK) was used to obtain mass spectra. A Perkin-Elmer CHN-2400 analyzer (Leco, St. Joseph, MI, USA) was used to conduct elemental microanalyses. The key precursor **3** was prepared as previously reported in the literature [24].

Biological methods. On medium potato dextrose agar (PDA), which involves a infusion of 200 g potatoes, 6 g dextrose and 15 g agar, the antimicrobial assay was carried out. Filter paper discs of uniform size (6 mm in diameter, with three discs for each compound) were carefully placed on an inoculated agar surface after being impregnated with an equivalent volume (10 µL) of dissolved compounds at concentrations of 500 and 1000 mg/mL in DMF. This was followed by 36 h of incubation at 27 °C for bacteria and 48 h at 24 °C for fungi. At dosages of 500 and 1000 mg/mL, the average diameter of the inhibitory zones surrounding the discs for each tested compound was measured in millimeters [27].

Mammalian HepG-2 and HCT-116 cell lines were obtained from the American Type Culture Collection (ATCC, Rockville, MD, USA). Using the HepG2 and HCT-116 human cancer cell lines, the 3-(4,5-dimethyl-2-thiazolyl)-2,5-diphenyl-2H-tetrazolium bromide (MTT) method was utilized to assess the anticancer efficacy of the synthesized compounds [28]. Before the MTT experiment, cells were placed in a 96-well sterile microplate (5 × 10^4^ cells/well), and each of the tested compounds at various dosages in DMSO or doxorubicin (a positive control) was incubated at 37 °C for 48 h in a serum-free medium. Following four hours of medium incubation, each well was treated with 40 µL of MTT (2.5 mg/mL) and another four hours of incubation. The purple formazan dye crystals were rendered soluble by adding 200 µL of DMSO. The absorbance was measured at 590 nm using a Spectra Max Paradigm Multi-Mode microplate reader (SunRise, TECAN, Inc., Morrisville, NC, USA). Relative cell viability was expressed as the mean percentage of viable cells compared with the untreated control cells. Each experiment was conducted three times. Using the GraphPad Prism program, the dose response curve graphic plots for each concentration were used to calculate the values of IC_50_, or the concentration required to cause detrimental effects in 50% of intact cells.

### 3.2. General Procedure for Synthesis of Heteroannulated Compounds ***4***–***14***

A mixture of compound **3** (0.34 g, 1 mmol) and carbon nucleophilic reagents, namely, 1,3-cyclohexanedione, dimedone, pyrazolidine-3,5-dione, 5-phenyl-2,4-dihydro-3*H*-pyrazol-3-one, 5-methyl-2-phenyl-2,4-dihydro-3H-pyrazol-3-one, 1,3-thiazolidine-2,4-dione, 2-(phenylimino)-1,3-thiazolidin-4-one, barbituric acid, thiobarbituric acid, 5-amine-3-methyl-1*H*-pyrazole and 6-amino-1,3-dimethyluracil (1 mmol), in *n*-butanol (10 mL) containing TEA (0.1 mL, 1 mmol) was heated under reflux for 30 min. The solids formed were filtered and crystallized from the proper solvent.

4,14-Dimethoxy-12-methyl-9,10-dihydro-6*H*-furo[3″,2″:6′,7′]chromeno[3′,4′:4,5]furo[3,2-*b*]quinoline-6,11(8*H*)-dione (**4**)

Crystallized from DMF/EtOH as yellow crystals, m.p. > 300 °C, yield (0.31 g, 74%). IR (KBr, cm^−1^): 3092 (CH_arom._), 2984, 2947 (CH_aliph._), 1716 (C=O_α-pyrone_), 1678 (C=O_quinolinone_), 1610 (C=N), 1594 (C=C). ^1^H-NMR (DMSO-*d*_6_, δ, 400 MHz): 2.02 (t, 2H, *J* = 6.4 Hz, CH_2_), 2.24 (s, 3H, CH_3_), 2.72 (t, 2H, *J* = 6.4 Hz, CH_2_), 3.12 (t, 2H, *J* = 6.4 Hz, CH_2_), 3.93 (s, 3H, OCH_3_), 4.03 (s, 3H, OCH_3_), 7.31 (d, 1H, *J* = 2.4 Hz, H-3_furan_), 8.03 (d, 2H, *J* = 2.4 Hz, H-2_furan_). ^13^C-NMR (DMSO-*d*_6_, δ, 100 MHz): 17.3 (CH_3_), 22.0 (CH_2_), 32.2 (CH_2_), 36.4 (CH_2_), 58.5 (OCH_3_), 59.4 (OCH_3_), 104.0, 106.1, 111.0, 114.8, 121.3, 128.6, 133.9, 141.1, 143.0, 144.8, 146.0, 147.2, 149.4, 151.8, 154.6, 164.3 (OC=O), 192.6 (C=O_quinoline_). Mass spectrum (*m*/*z*, *I* %): 419 (56), 391 (100), 361 (52), 305 (38), 277 (30), 231 (24), 174 (18), 158 (47), 133 (24), 108 (23), 77 (28), 64 (13). Anal. Calcd for C_23_H_17_NO_7_ (419.38): C, 65.87; H, 4.09; N, 3.34%. Found: C, 65.70; H, 4.01; N, 3.32%.

4,14-Dimethoxy-9,9,12-trimethyl-9,10-dihydro-6*H*-furo[3″,2″:6′,7′]chromeno[3′,4′:4,5]furo[3,2-*b*]quinoline-6,11(8*H*)-dione (**5**)

Crystallized from DMF as pale-yellow crystals, m.p. > 300 °C, yield (0.32 g, 72%). IR (KBr, cm^−1^): 2968, 2946, 2928 (CH_aliph._), 1712 (C=O_α-pyrone_), 1681 (C=O_quinolinone_), 1614 (C=N), 1593 (C=C). ^1^H-NMR (DMSO-*d*_6_, δ, 400 MHz): 1.03 (s, 6H, 2CH_3_), 2.31 (s, 3H, CH_3_), 2.74 (s, 2H, CH_2_), 2.92 (s, 2H, CH_2_), 3.92 (s, 3H, OCH_3_), 4.03 (s, 3H, OCH_3_), 7.15 (d, 1H, *J* = 2.0 Hz, H-3_furan_), 7.91 (d, 1H, *J* = 2.0 Hz, H-2_furan_). ^13^C-NMR (DMSO-*d*_6_, δ, 100 MHz): 12.9 (2CH_3_), 15.3 (CMe_2_), 17.8 (CH_3_), 31.9 (CH_2_), 36.7 (CH_2_), 59.8 (OCH_3_), 60.2 (OCH_3_), 105.4, 106.1, 110.5, 115.2, 121.3, 128.2, 132.5, 141.4, 143.1, 143.8, 145.2, 146.5, 148.3, 150.5, 154.7, 164.5 (OC=O), 191.8 (C=O_quinolinone_). Mass spectrum (*m*/*z*, *I* %): 447 (100), 419 (45), 359 (36), 318 (28), 277 (35), 232 (26), 174 (29), 134 (15), 107 (27), 92 (36), 77 (43), 64 (13). Anal. Calcd for C_25_H_21_NO_7_ (447.44): C, 67.11; H, 4.73; N, 3.13%. Found: C, 66.95; H, 4.69; N, 3.02%.

4,13-Dimethoxy-11-methyl-8,9-dihydro-6*H*,10*H*-furo[3″,2″:6′,7′]chromeno[3′,4′:4,5] furo[3,2-*b*]pyrazolo[4,3-*e*]pyridine-6,10-dione (**6**)

Crystallized from xylene as yellow crystals, m.p. > 300 °C, yield (0.28 g, 69%). IR (KBr, cm^−1^): 3379, 3228 (2NH), 2973, 2921 (CH_aliph._), 1723 (C=O_α-pyrone_), 1679 (C=O_pyrazolone_), 1614 (C=N), 1584 (C=C). ^1^H-NMR (DMSO-*d*_6_, δ, 400 MHz): 2.27 (s, 3H, CH_3_), 3.92 (s, 3H, OCH_3_), 3.97 (s, 3H, OCH_3_), 7.22 (d, 1H, *J* = 2.4 Hz, H-3_furan_), 8.03 (d, 2H, *J* = 2.4 Hz, H-2_furan_), 11.48 (bs, 2H, 2NH vanished with D_2_O). ^13^C NMR (DMSO-d_6_, δ, 100 MHz): 17.9 (CH_3_), 59.7 (OCH_3_), 60.6 (OCH_3_), 104.6, 106.1, 110.5, 115.0, 122.8, 124.1, 128.4, 140.6, 142.8, 144.5, 146.1, 147.8, 149.1, 152.5, 155.1, 163.3 (OC=O), 170.6 (C=O_pyrazolone_). Mass spectrum (*m*/*z*, *I* %): 407 (100), 364 (71), 334 (44), 278 (52), 235 (24), 159 (37), 108 (32), 92 (51), 77 (19), 64 (13). Anal. Calcd for C_20_H_13_N_3_O_7_ (407.33): C, 58.97; H, 3.22; N, 10.32%. Found: C, 58.76; H, 3.20; N, 10.17%.

4,13-Dimethoxy-11-methyl-10-phenyl-6*H*,8*H*-furo[3″,2″:6′,7′]chromeno[3′,4′:4,5]furo[3,2-*b*]pyrazolo[4,3-*e*]pyridin-6-one (**7**)

Crystallized from toluene as pale -yellow crystals, m.p. > 300 °C, yield (0.34 g, 73%). IR (KBr, cm^−1^): 3226 (NH), 3032 (CH_arom._), 2965, 2937 (CH_aliph._), 1726 (C=O_α-pyrone_), 1613 (C=N), 1590 (C=C). ^1^H-NMR (DMSO-*d*_6_, δ, 400 MHz): 2.29 (s, 3H, CH_3_), 3.91 (s, 3H, OCH_3_), 4.03 (s, 3H, OCH_3_), 7.21 (d, 1H, *J* = 2.4 Hz, H-3_furan_), 7.40–7.49 (m, 5H, Ph-H), 7.96 (d, 1H, *J* = 2.4 Hz, H-2_furan_), 10.75 (s, 1H, NH vanished with D_2_O). Mass spectrum (*m*/*z*, *I* %): 467 (100), 407 (48), 330 (39), 234 (42), 190 (18), 158 (41), 134 (15), 108 (25), 92 (31), 77 (26), 64 (14). Anal. Calcd for C_26_H_17_N_3_O_6_ (467.43): C, 66.81; H, 3.67; N, 8.99%. Found: C, 66.74; H, 3.43; N, 8.86%.

4,13-Dimethoxy-10,11-dimethyl-8-phenyl-6*H*,8*H*-furo[3″,2″:6′,7′]chromeno[3′,4′:4,5] furo[3,2-*b*]pyrazolo[4,3-*e*]pyridin-6-one (**8**)

Crystallized from AcOH/H_2_O as yellow crystals, m.p. > 300 °C, yield (0.36 g, 75%). IR (KBr, cm^−1^): 3023 (CH_arom._), 2954, 2926 (CH_aliph._), 1718 (C=O_α-pyrone_), 1615 (C=N), 1584 (C=C). ^1^H-NMR (DMSO-*d*_6_, δ, 400 MHz): 2.03 (s, 3H, CH_3_), 2.25 (s, 3H, CH_3_), 3.88 (s, 3H, OCH_3_), 3.97 (s, 3H, OCH_3_), 7.19 (d, 1H, *J* = 2.4 Hz, H-3_furan_), 7.34–7.48 (m, 5H, Ph-H), 8.05 (d, 1H, *J* = 2.4 Hz, H-2_furan_). Mass spectrum (*m*/*z*, *I* %): 481 (25), 404 (57), 359 (51), 318 (43), 274 (29), 238 (17), 175 (32), 158 (20), 133 (34), 108 (47), 77 (100), 65 (35). Anal. Calcd for C_27_H_19_N_3_O_6_ (481.46): C, 67.36; H, 3.98; N, 8.73%. Found: C, 67.31; H, 3.83; N, 8.64%.

4,13-Dimethoxy-11-methyl-6*H*-furo[3″,2″:6′,7′]chromeno[3′,4′:4,5]furo[3,2-*b*]thiazolo[5,4-*e*]pyridine-6,9(8*H*)-dione (**9**)

Crystallized from isobutanol as orange crystals, m.p. > 300 °C, yield (0.30 g, 71%). IR (KBr, cm^−1^): 3239 (NH), 2974, 2925 (CH_aliph._), 1725 (C=O_α-pyrone_), 1673 (C=O_thiazole_), 1610 (C=N), 1576 (C=C). ^1^H-NMR (DMSO-*d*_6_, δ, 400 MHz): 2.30 (s, 3H, CH_3_), 3.91 (s, 3H, OCH_3_), 4.03 (s, 3H, OCH_3_), 7.20 (d, 1H, *J* = 2.0 Hz, H-3_furan_), 7.96 (d, 1H, *J* = 2.4 Hz, H-2_furan_), 11.19 (s, 1H, NH vanished with D_2_O). ^13^C NMR (DMSO-d_6_, δ, 100 MHz): 17.8 (CH_3_), 59.3 (OCH_3_), 60.3 (OCH_3_), 105.3, 106.6, 110.4, 115.9, 122.2, 128.4, 132.5, 141.6, 142.9, 144.0, 144.9, 146.8, 147.6, 151.3, 154.9, 164.7 (OC=O), 171.3 (C=O_thiazole_). Mass spectrum (*m*/*z*, *I* %): 424 (100), 366 (64), 325 (41), 281 (37), 236 (24), 173 (26), 158 (17), 108 (71), 92 (39), 77 (28), 64 (9). Anal. Calcd for C_20_H_12_N_2_O_7_S (424.38): C, 56.60; H, 2.85; N, 6.60; S, 7.56%. Found: C, 56.39; H, 2.74; N, 6.41; S, 7.50%.

4,13-Dimethoxy-11-methyl-9-(phenylimino)-8,9-dihydro-6*H*-furo[3″,2″:6′,7′] chromeno[3′,4′:4,5]furo[3,2-*b*]thiazolo[5,4-*e*]pyridin-6-one (**10**)

Crystallized from DMF/H_2_O as pale brown crystals, m.p. > 300 °C, yield (0.33 g, 66%). IR (KBr, cm^−1^): 3215 (NH), 3043 (CH_arom._), 2961, 2927 (CH_aliph._), 1720 (C=O_α-pyrone_), 1607 (C=N), 1579 (C=C). ^1^H-NMR (DMSO-*d*_6_, δ, 400 MHz): 2.22 (s, 3H, CH_3_), 3.89 (s, 3H, OCH_3_), 3.98 (s, 3H, OCH_3_), 7.27 (d, 1H, *J* = 2.4 Hz, H-3_furan_), 7.48–7.59 (m, 5H, Ph-H), 7.96 (d, 1H, *J* = 2.4 Hz, H-2_furan_), 10.31 (s, 1H, NH vanished with D_2_O). Mass spectrum (*m*/*z*, *I* %): 499 (69), 439 (47), 362 (50), 321 (42), 261 (37), 228 (42), 159 (34), 133 (21), 107 (100), 92 (67), 77 (36), 64 (15). Anal. Calcd for C_26_H_17_N_3_O_6_S (499.49): C, 62.52; H, 3.43; N, 8.41; S, 6.42%. Found: C, 62.36; H, 3.40; N, 8.23; S, 6.29%.

4,14-Dimethoxy-12-methyl-6*H*-furo[3′′′,2′′′:6″,7″]chromeno[3″,4″:4′,5′]furo[2′,3′:5,6] pyrido[2,3-*d*]pyrimidine-6,9,11(8*H*,10*H*)-trione (**11**)

Crystallized from gl. AcOH as yellow crystals, m.p. > 300 °C, yield (0.32 g, 74%). IR (KBr, cm^−1^): 3314, 3249 (NH), 2959, 2941 (CH_aliph._), 1722 (C=O_α-pyrone_), 1691 (C=O_pyrimidine_), 1617 (C=N), 1582 (C=C). ^1^H-NMR (DMSO-*d*_6_, δ, 400 MHz): 2.31 (s, 3H, CH_3_), 3.91 (s, 3H, OCH_3_), 3.98 (s, 3H, OCH_3_), 7.25 (d, 1H, *J* = 2.0 Hz, H-3_furan_), 8.03 (d, 1H, *J* = 2.0 Hz, H-2_furan_), 10.75 (bs, 2H, 2NH vanished with D_2_O). ^13^C NMR (DMSO-d_6_, δ, 100 MHz): 17.4 (CH_3_), 59.3 (OCH_3_), 60.2 (OCH_3_), 104.7, 106.1, 110.5, 114.9, 124.3, 128.7, 133.1, 140.5, 143.1, 144.7, 145.0, 146.2, 148.5, 150.3, 155.8, 164.4 (OC=O), 171.3 (C=O_pyrimidine_), 173.6 (C=O_pyrimidine_). Mass spectrum (*m*/*z*, *I* %): 435 (100), 379 (63), 334 (56), 290 (53), 248 (39), 223 (27), 158 (14), 134 (16), 93 (28), 77 (25), 64 (8). Anal. Calcd for C_21_H_13_N_3_O_8_ (435.34): C, 57.94; H, 3.01; N, 9.65%. Found: C, 57.76; H, 2.89; N, 9.46%.

4,14-Dimethoxy-12-methyl-9-thioxo-9,10-dihydro-6*H*-furo[3′′′,2′′′:6″,7″]chromeno[3″,4″:4′,5′]furo[2′,3′:5,6]pyrido[2,3-*d*]pyrimidine-6,11(8*H*)-dione (**12**)

Crystallized from DMF/H_2_O as yellow crystals, m.p. > 300 °C, yield (0.35 g, 78%). IR (KBr, cm^−1^): 3336, 3261 (NH), 2968, 2932 (CH_aliph._), 1716 (C=O_α-pyrone_), 1687 (C=O_pyrimidine_), 1611 (C=N), 1583 (C=C), 1236 (C=S). ^1^H-NMR (DMSO-*d*_6_, δ, 400 MHz): 2.27 (s, 3H, CH_3_), 3.92 (s, 3H, OCH_3_), 4.01 (s, 3H, OCH_3_), 7.21 (d, 1H, *J* = 2.4 Hz, H-3_furan_), 7.96 (d, 1H, *J* = 2.4 Hz, H-2_furan_), 11.03 (bs, 2H, 2NH vanished with D_2_O). ^13^C NMR (DMSO-d_6_, δ, 100 MHz): 17.1 (CH_3_), 59.7 (OCH_3_), 60.3 (OCH_3_), 104.5, 106.4, 110.0, 115.2, 124.6, 128.5, 132.4, 140.8, 143.2, 144.7, 145.2, 146.5, 148.8, 150.9, 155.4, 164.0 (OC=O), 171.7 (C=O_pyrimidine_), 185.2 (C=S). Mass spectrum (*m*/*z*, *I* %): 451 (100), 409 (71), 353 (26), 311 (37), 232 (43), 173 (31), 157 (22), 133 (18), 92 (56), 77 (38), 65 (13). Anal. Calcd for C_21_H_13_N_3_O_7_S (451.41): C, 55.88; H, 2.90; N, 9.31; S, 7.10%. Found: C, 55.67; H, 2.83; N, 9.18; S, 7.02%.

4,13-Dimethoxy-10,11-dimethyl-6*H*,8*H*-furo[3″,2″:6′,7′]chromeno[3′,4′:4,5]furo[3,2-*b*]pyrazolo[4,3-*e*]pyridin-6-one (**13**)

Crystallized from DMF as pale yellow crystals, m.p. > 300 °C, yield (0.32 g, 79%). IR (KBr, cm^−1^): 3216 (NH), 2969, 2942, 2938 (CH_aliph._), 1721 (C=O_α-pyrone_), 1615 (C=N), 1580 (C=C). ^1^H-NMR (DMSO-*d*_6_, δ, 400 MHz): 2.20 (s, 3H, CH_3_), 2.31 (s, 3H, CH_3_), 3.91 (s, 3H, OCH_3_), 4.02 (s, 3H, OCH_3_), 7.25 (d, 1H, *J* = 2.4 Hz, H-3_furan_), 7.94 (d, 1H, *J* = 2.4 Hz, H-2_furan_), 10.85 (s, 1H, NH vanished with D_2_O). ^13^C NMR (DMSO-d_6_, δ, 100 MHz): 16.3 (CH_3_), 17.3 (CH_3_), 58.7 (OCH_3_), 59.8 (OCH_3_), 105.9, 106.2, 110.9, 114.8, 123.1, 128.7, 133.2, 140.8, 143.7, 144.8, 145.5, 147.2, 148.5, 149.7, 151.7, 154.6, 163.8 (OC=O). Mass spectrum (*m*/*z*, *I* %): 405 (100), 304 (48), 260 (39), 231 (42), 173 (20), 157 (24), 133 (46), 108 (51), 77 (35), 65 (9). Anal. Calcd for C_21_H_15_N_3_O_6_ (405.36): C, 62.22; H, 3.73; N, 10.37%. Found: C, 62.13; H, 3.55; N, 10.21%.

4,14-Dimethoxy-8,10,12-trimethyl-6*H*-furo[3′′′,2′′′:6″,7″]chromeno[3″,4″:4′,5′]furo[2′,3′:5,6]pyrido[2,3-*d*]pyrimidine-6,9,11(8*H*,10*H*)-trione (**14**)

Crystallized from gl. AcOH as yellow crystals, m.p. > 300 °C, yield (0.38 g, 77%). IR (KBr, cm^−1^): 2994, 2976, 2951 (CH_aliph._), 1723 (C=O_α-pyrone_), 1704, 1689 (2C=O_pyrimidine_), 1607 (C=N), 1574 (C=C). ^1^H-NMR (DMSO-*d*_6_, δ, 400 MHz): 2.35 (s, 3H, CH_3_), 3.06 (s, 3H, NCH_3_), 3.19 (s, 3H, NCH_3_), 3.92 (s, 3H, OCH_3_), 4.06 (s, 3H, OCH_3_), 7.23 (d, 1H, *J* = 2.4 Hz, H-3_furan_), 8.02 (d, 1H, *J* = 2.4 Hz, H-2_furan_). ^13^C NMR (DMSO-d_6_, δ, 100 MHz): 16.5 (CH_3_), 26.4 (NCH_3_), 29.4 (NCH_3_), 59.7 (OCH_3_), 60.6 (OCH_3_), 105.4, 106.5, 110.9, 114.7, 121.8, 129.9, 141.1, 141.9, 142.1, 146.5, 147.1, 147.6, 149.8, 152.1, 154.6, 165.2 (OC=O), 172.4 (C=O_pyrimidine_), 175.4 (C=O_pyrimidine_). Mass spectrum (*m*/*z*, *I* %): 463 (58), 435 (100), 392 (59), 348 (50), 303 (42), 223 (27), 162 (22), 135 (19), 108 (15), 94 (41), 77 (68), 65 (24). Anal. Calcd for C_23_H_17_N_3_O_8_ (463.41): C, 59.61; H, 3.70; N, 9.07%. Found: C, 59.47; H, 3.65; N, 8.84%.

## 4. Conclusions

The present study provided a useful approach for generating the novel heteroannulated furochromenofuropyridines through Friedländer reactions of the starting compound **3** with active methylene nucleophiles, namely, 1,3-cyclohexanediones, pyrazolones, 1,3-thiazolidinones, barbituric acids, 5-amine-3-methyl-1*H*-pyrazole and 6-amino-1,3-dimethyluracil. The synthesized compounds showed diverse efficiency against the microbial strains, where compound **3** is the most effective candidate towards all types of microorganisms. The compounds derived from the heteroannulation of compound **3** with thiazolopyridines (**9** and **10**) and pyridopyrimidne moieties (**11**, **12** and **14**) showed excellent cytotoxic activity against both types of the studied cancer cell lines. Meanwhile, annulation with quinolines (**4**,**5**) and pyrazolopyridines (**6**–**8** and **13**) led to good cytotoxic activity.

## Figures and Tables

**Table 1:** In vitro antimicrobial activity for compounds **3**–**14** by disc diffusion assay, at 500 and 1000 μg/mL.

No.	Zone Diameter (mm) *											
	Gram-Positive Bacteria				Gram-Negative Bacteria				Yeasts and Fungi			
Compd. No.	*Staphylococcus aureus*		*Bacillus subtilis*		*Salmonella typhimurium*		*Escherichia coli*		*Candida albicans*		*Asperigillus fumigatus*	
	1000 μg/mL	500 μg/mL	1000 μg/mL	500 μg/mL	1000 μg/mL	500 μg/mL	1000 μg/mL	500 μg/mL	1000 μg/mL	500 μg/mL	1000 μg/mL	500 μg/mL
**3**	26 H	19 H	24 H	18 H	26 H	19 H	28 H	20 H	27 H	22 H	27 H	18 H
**4**	18 I	12 I	21 I	14 I	19 I	14 I	17 I	13 I	15 I	12 I	19 I	14 I
**5**	17 I	13 I	17 I	11 I	14 I	10 I	15 I	11 I	18 I	15 I	18 I	12 I
**6**	16 I	10 I	15 I	12 I	27 H	21 H	26 H	18 H	27 H	21 H	26 H	19 H
**7**	19 I	12 I	13 I	9 I	25 H	20 H	28 H	22 H	24 H	19 H	25 H	18 H
**8**	18 I	13 I	15 I	10 I	26 H	20 H	25 H	19 H	27 H	20 H	28 H	20 H
**9**	20 I	13 I	14 I	11 I	25 H	18 I	27 H	20 H	22 I	18 I	19 I	16 I
**10**	14 I	10 I	16 I	11 I	28 H	21 H	25 H	18 H	20 I	16 I	17 I	12 I
**11**	25 H	18 H	27 H	20 H	20 I	15 I	17 I	12 I	17 I	13 I	19 I	14 I
**12**	29 H	21 H	30 H	21 H	18 I	13 I	16 I	11 I	16 I	12 I	14 I	10 I
**13**	17 I	13 I	20 I	16 I	27 H	20 H	30 H	22 H	26 H	19 H	27 H	21 H
**14**	26 H	20 H	25 H	19 H	20 I	15 I	16 I	10 I	17 I	12 I	17 I	13 I
**S**	35	26	35	25	36	28	38	27	35	28	37	26

* Calculated from three values. H = high activity (zone diameter ≥ 2/3 zone diameter of standard drug), I = intermediate activity (zone diameter < 2/3 and ≥ 1/3 zone diameter of standard drug). **S**: standard antibiotics chloramphenicol, cephalothin and cycloheximide were utilized for Gram-positive bacteria, Gram-negative bacteria, and fungi, respectively.

**Table 2:** In vitro anticancer efficiency (IC_50_) of compounds **3**–**14** against HCT-116 and HepG2 cell lines.

Compound No.	IC_50_ (µg/mL) *	
	HCT-116	HepG2
**3**	31.70 ± 1.25	29.53 ± 1.02
**4**	17.36 ± 0.91	18.76 ± 0.84
**5**	19.27 ± 0.65	14.37 ± 0.65
**6**	18.83 ± 0.63	16.94 ± 0.75
**7**	14.26 ± 0.39	15.16 ± 0.32
**8**	19.21 ± 0.84	17.09 ± 0.65
**9**	8.44 ± 0.38	6.76 ± 0.21
**10**	9.30 ± 0.57	8.52 ± 0.37
**11**	9.15 ± 0.46	9.41 ± 0.61
**12**	8.17 ± 0.43	7.87 ± 0.52
**13**	17.53 ± 0.48	13.82 ± 0.96
**14**	7.56 ± 0.67	5.96 ± 0.74
**Doxorubicin**	4.22 ± 0.18	4.58 ± 0.13

* IC_50_ (µg/mL): 21–50 (moderate), 11–20 (good) and 1–10 (excellent).

## Data Availability

Data are contained within the article and Supplementary Materials.

## Supplementary Materials

The following supporting information can be downloaded at: https://www.mdpi.com/article/10.3390/molecules29102319/s1.
