# Supplementary material for: Synthesis, Characterization and Antimicrobial and Anticancer Evaluations of Some Novel Heteroannulated Difuro[3,2-c:3′,2′-g]Chromenes
Source: Molecules. 2024 May 15;29(10):2319. doi: 10.3390/molecules29102319 (PMC11123756; doi:10.3390/molecules29102319)
Supplement: Supplementary file 1 [file molecules-29-02319-s001.zip › molecules-2959640-supplementary.pdf]

Synthesis, characterization, antimicrobial and anticancer evaluations of Some novel heteroannulated difuro[3,2-*c*:3',2'-*g*]chromenes

Najla A. Alshaye,[1] Magdy A. Ibrahim[*, 2] and Al-Shimaa Badran[2]

[1] Department of Chemistry, College of Science, Princess Nourah bint Abdulrahman University, P.O. Box 84428, Riyadh 11671, Saudi Arabia. naalshaye@pnu.edu.sa

[2] Department of Chemistry, Faculty of Education, Ain Shams University, Roxy, 11711, Cairo, Egypt

***** E-mail: magdy_ahmed1977@yahoo.com, Tel.: +2 01007887204 fax:+2 022581243

<u>Copies of IR, ^{1}H-NMR, ^{13}C-NMR and mass spectral data for the synthesized compounds:</u>

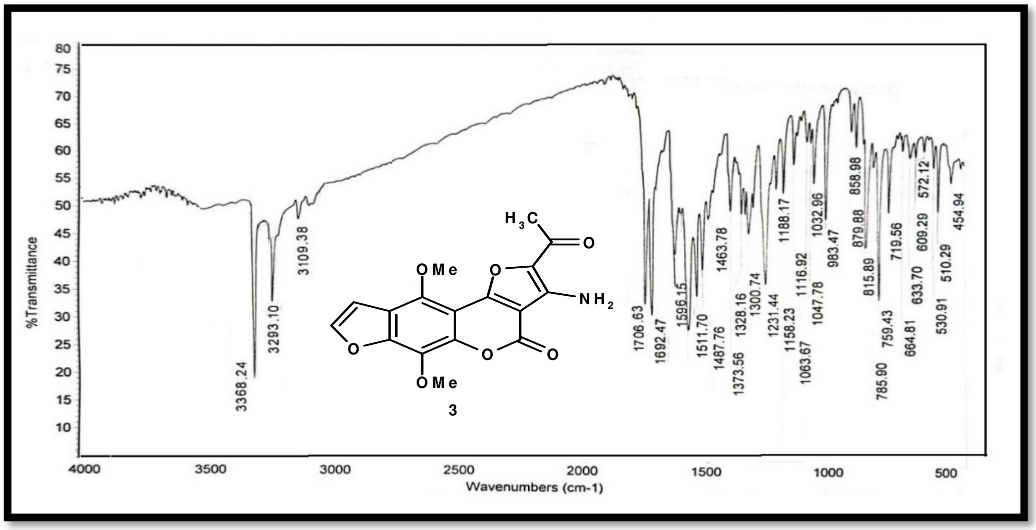

Figure S1: IR Spectrum of compound 3

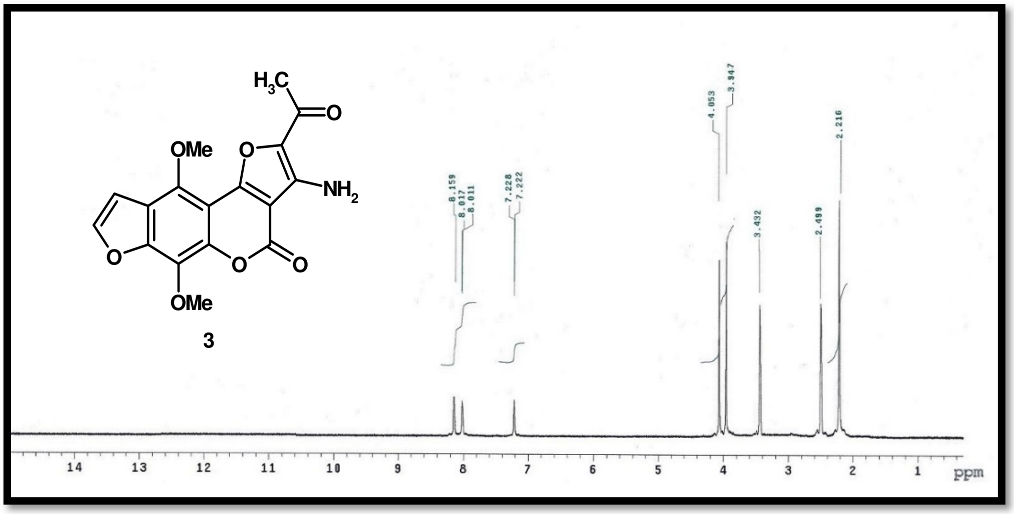

Figure S2 (DMSO-d_6, 400 MHz): ^{1}H NMR Spectrum of compound 3

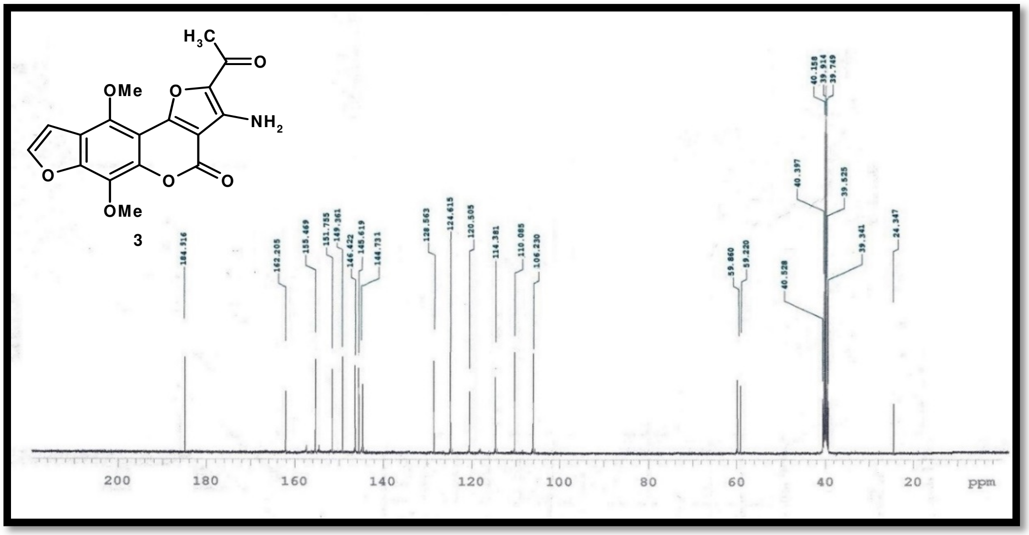

Figure S3 (DMSO-d_6, 100 MHz): ^{13}C NMR Spectrum of compound 3

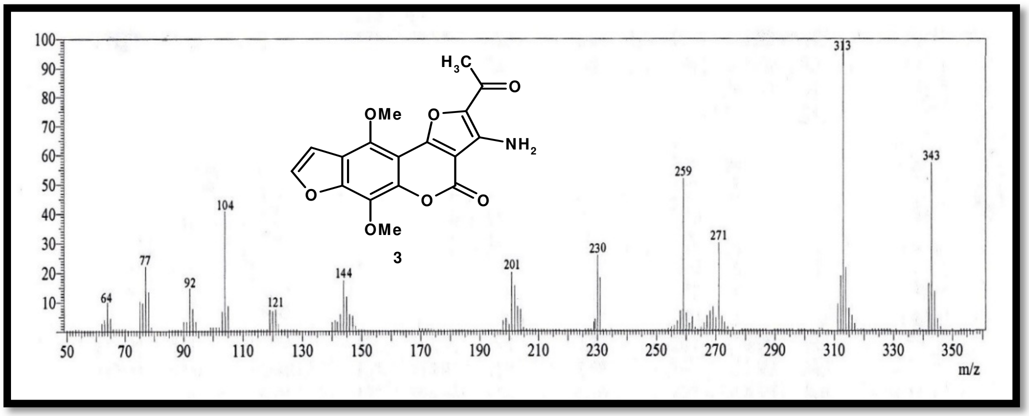

Figure S4: Mass Spectrum of compound 3

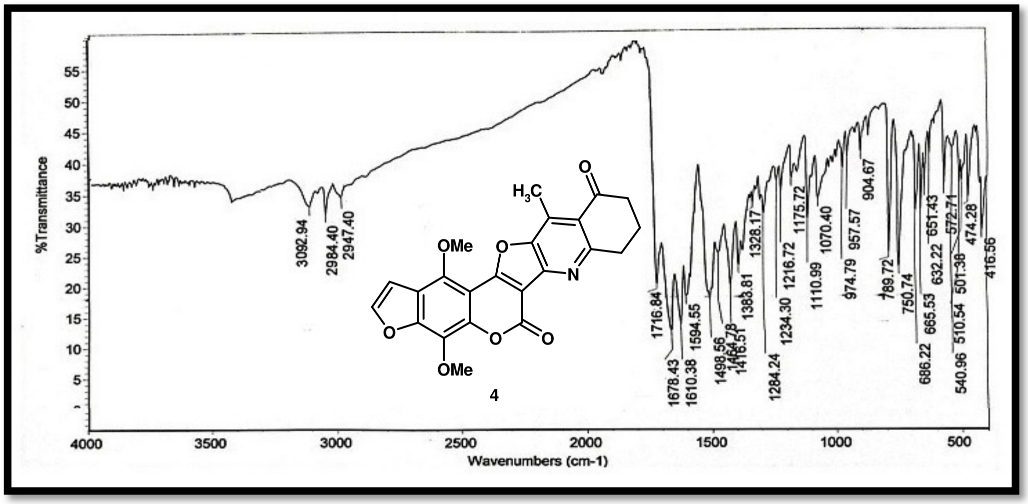

Figure S5: IR Spectrum of compound 4

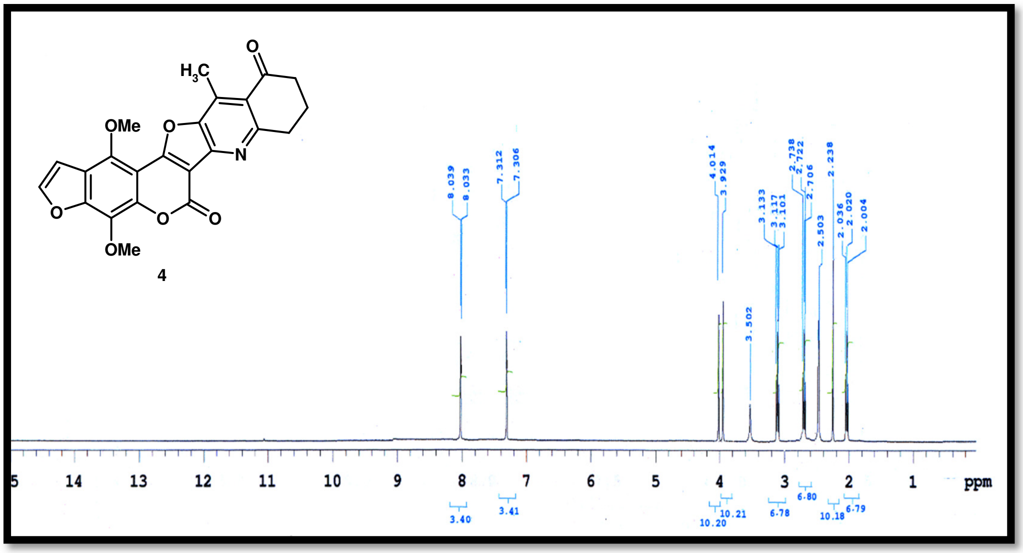

Figure S6 (DMSO-d_6, 400 MHz): ^{1}H NMR Spectrum of compound 4

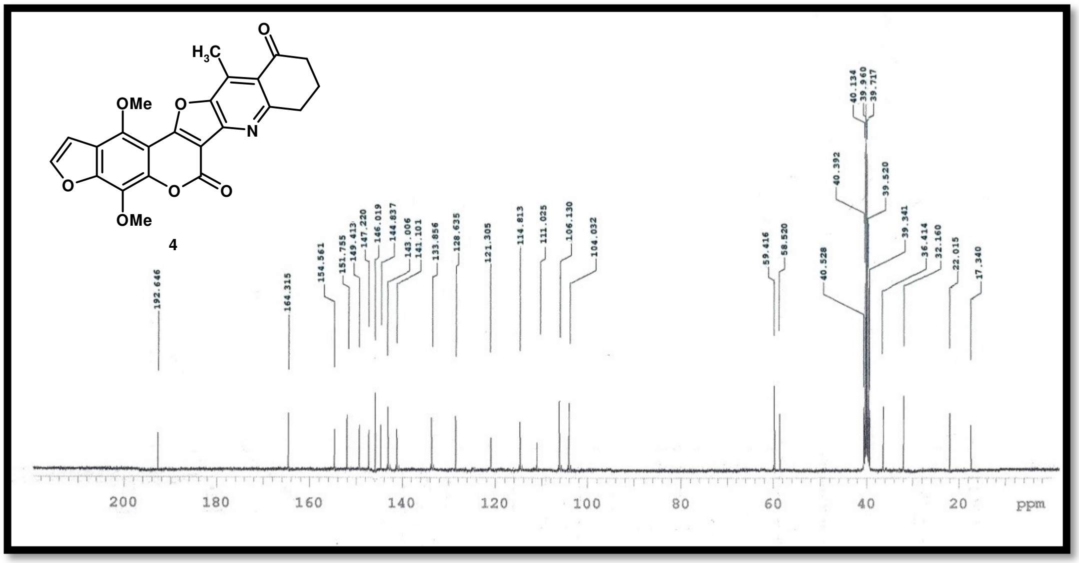

Figure S7 (DMSO-d_6, 100 MHz): ^{13}C NMR Spectrum of compound 4

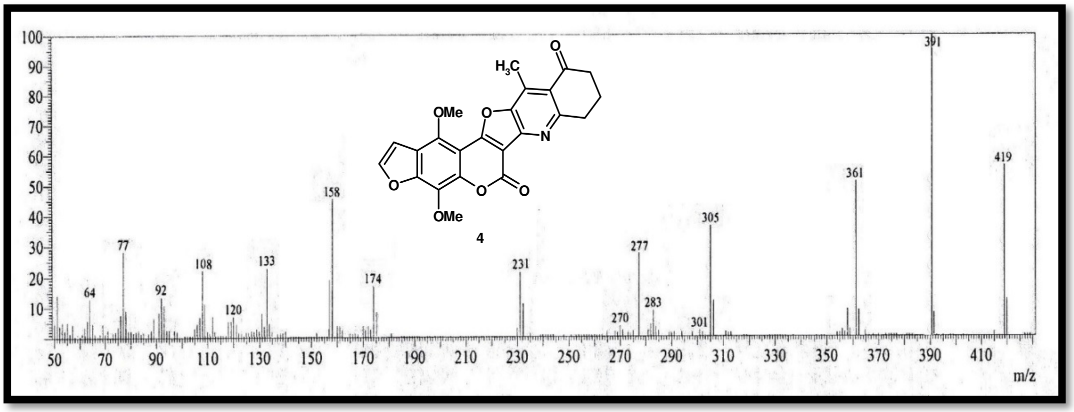

Figure S8: Mass Spectrum of compound 4

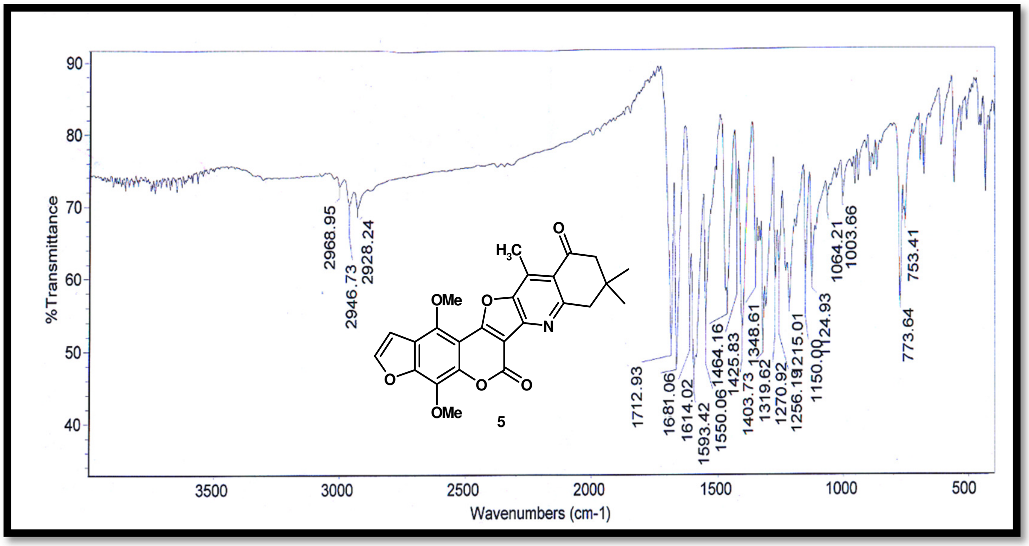

Figure S9: IR Spectrum of compound 5

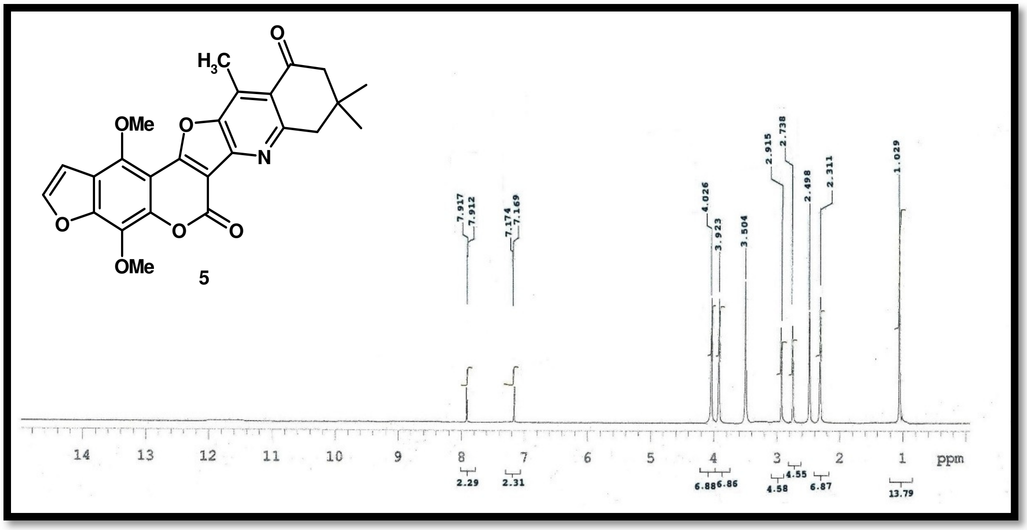

Figure S10 (DMSO-d_6, 400 MHz): ^{1}H NMR Spectrum of compound 5

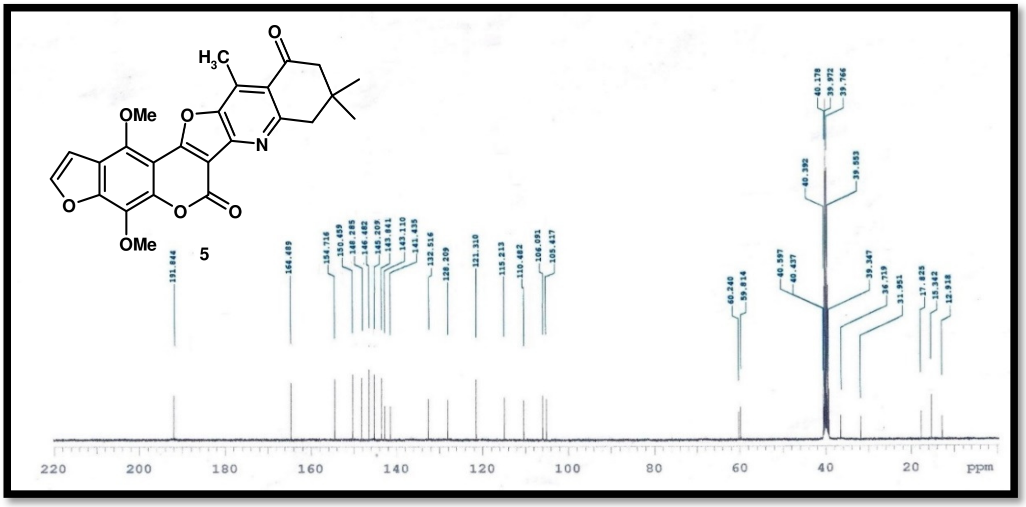

Figure S11 (DMSO-d_6, 100 MHz): ^{13}C NMR Spectrum of compound 5

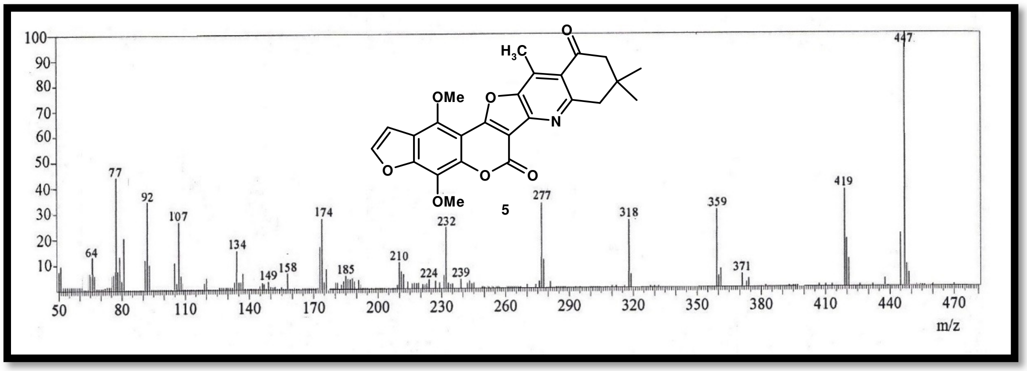

Figure S12: Mass Spectrum of compound 5

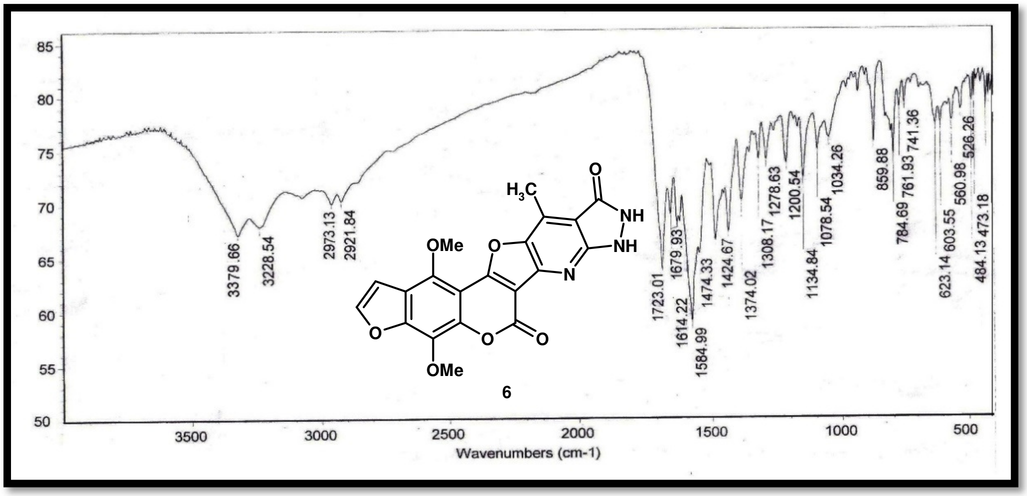

Figure S13: IR Spectrum of compound 6

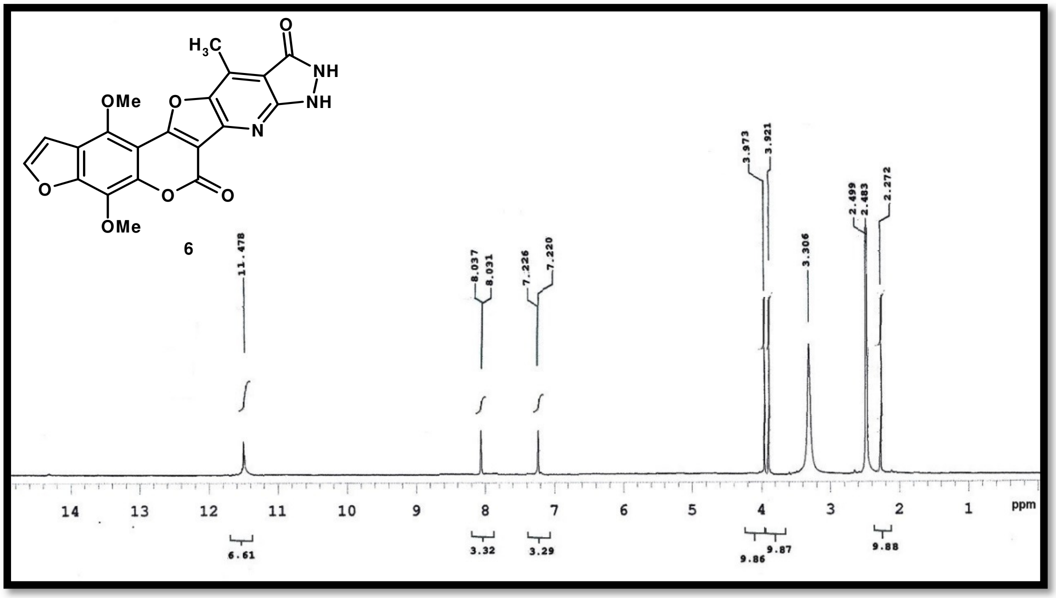

Figure S14 (DMSO-d_6, 400 MHz): ^{1}H NMR Spectrum of compound 6

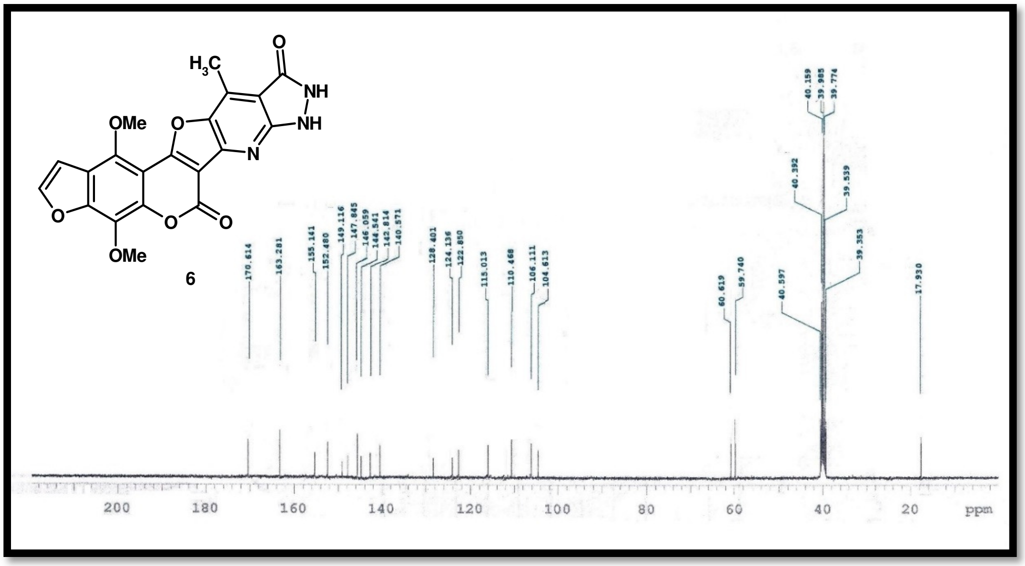

Figure S15 (DMSO-d_6, 100 MHz): ^{13}C NMR Spectrum of compound 6

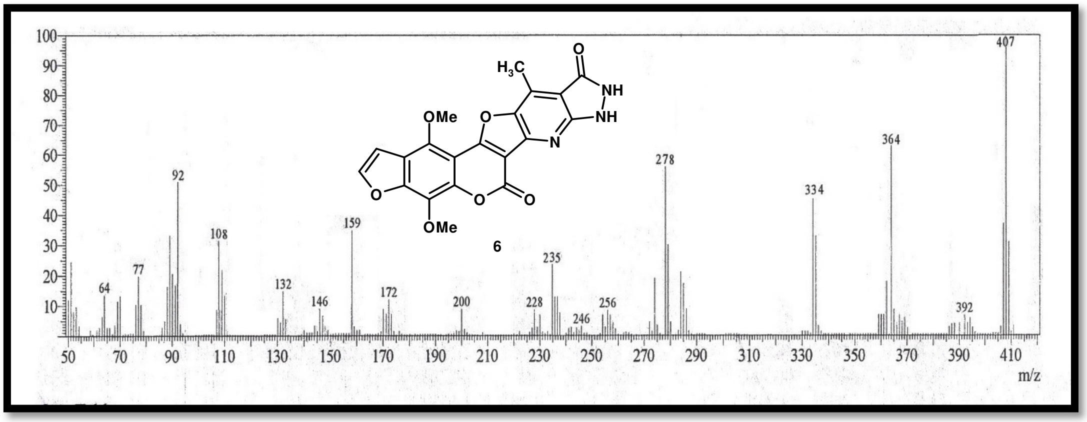

Figure S16: Mass Spectrum of compound 6

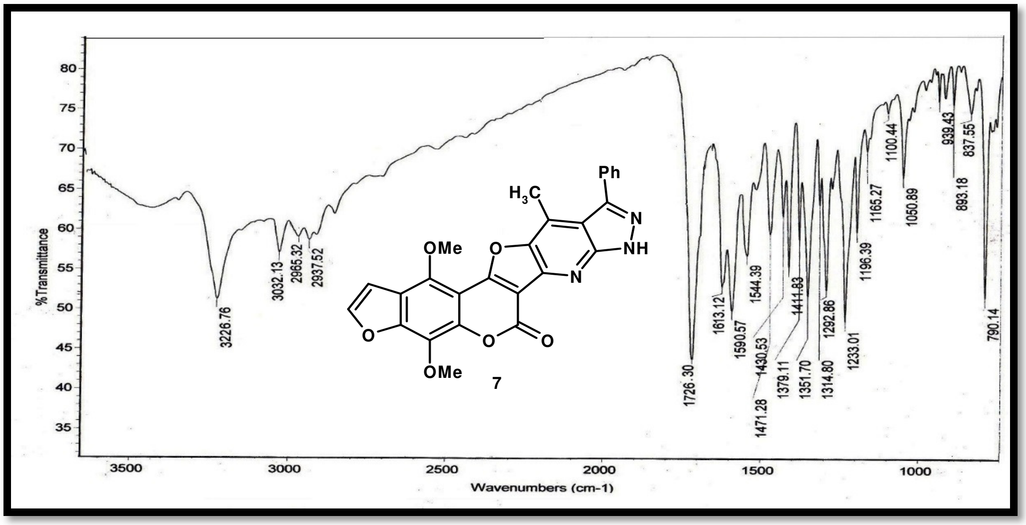

Figure S17: IR Spectrum of compound 7

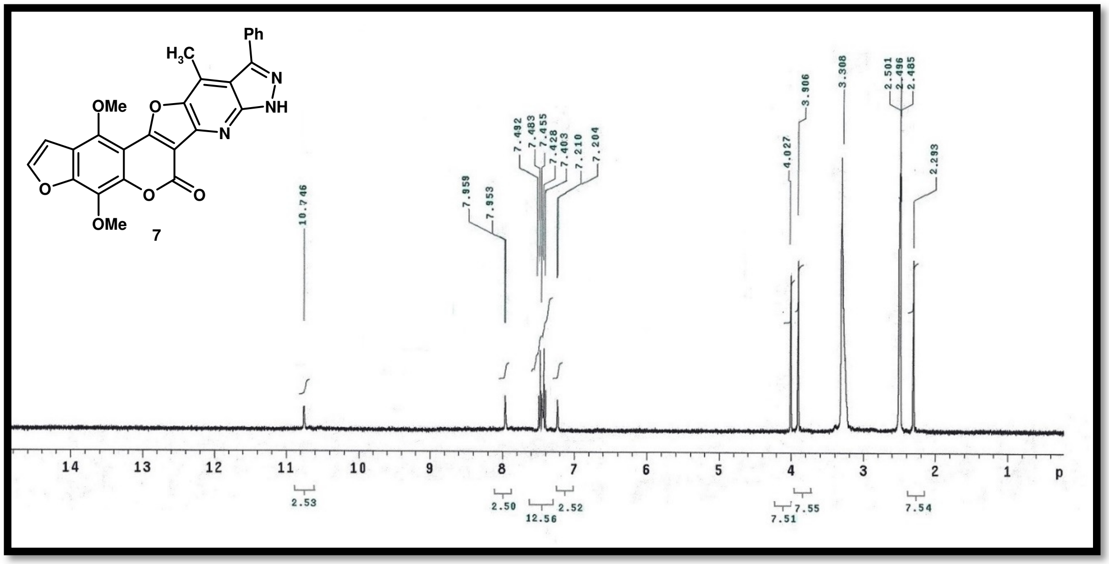

Figure S18 (DMSO-d_6, 400 MHz): ^{1}H NMR Spectrum of compound 7

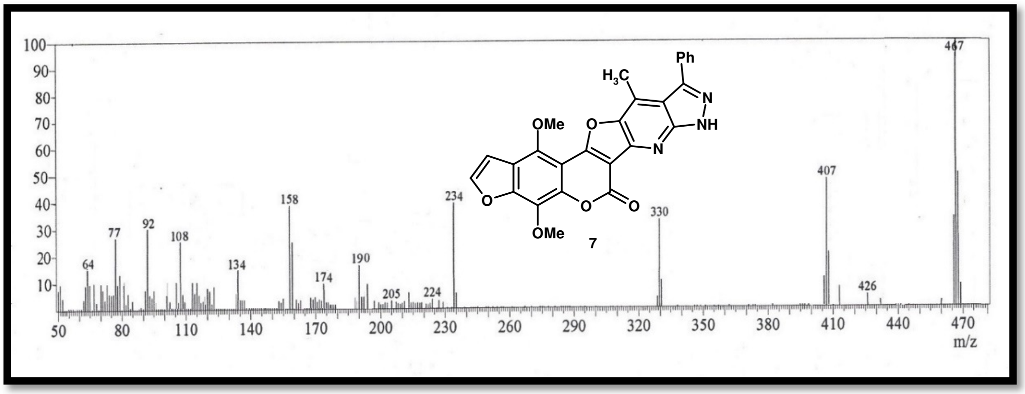

Figure S19: Mass Spectrum of compound 7

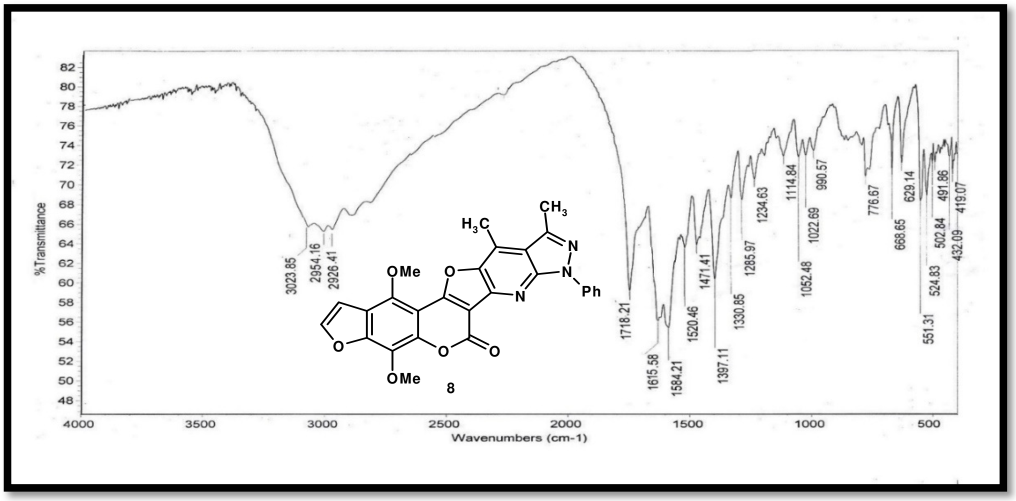

Figure S20: IR Spectrum of compound 8

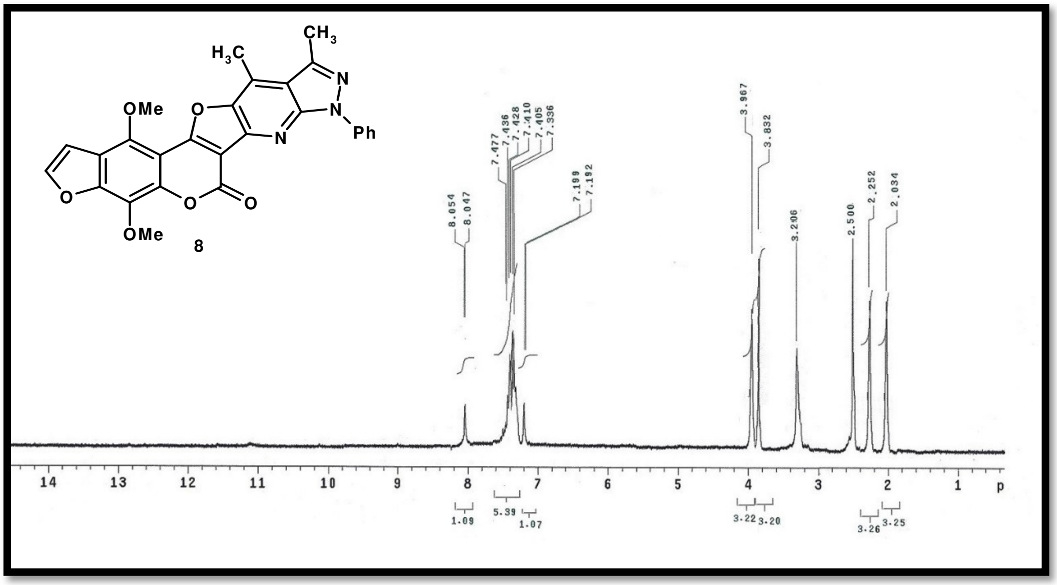

Figure S21 (DMSO-d_6, 400 MHz): ^{1}H NMR Spectrum of compound 8

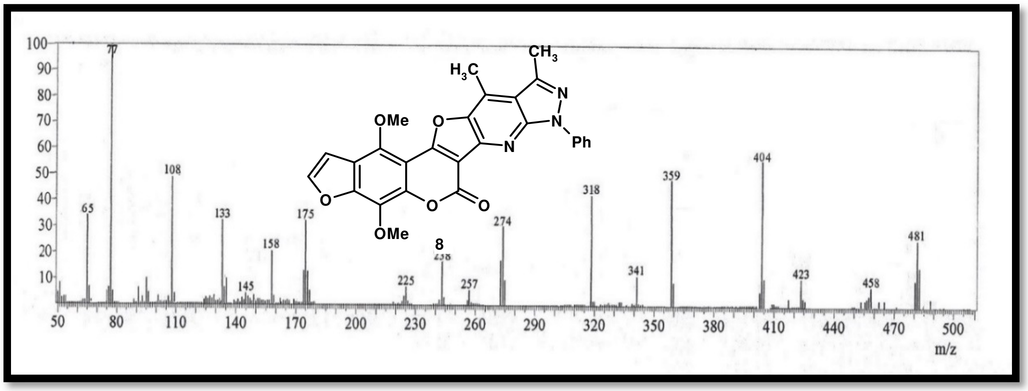

Figure S22: Mass Spectrum of compound 8

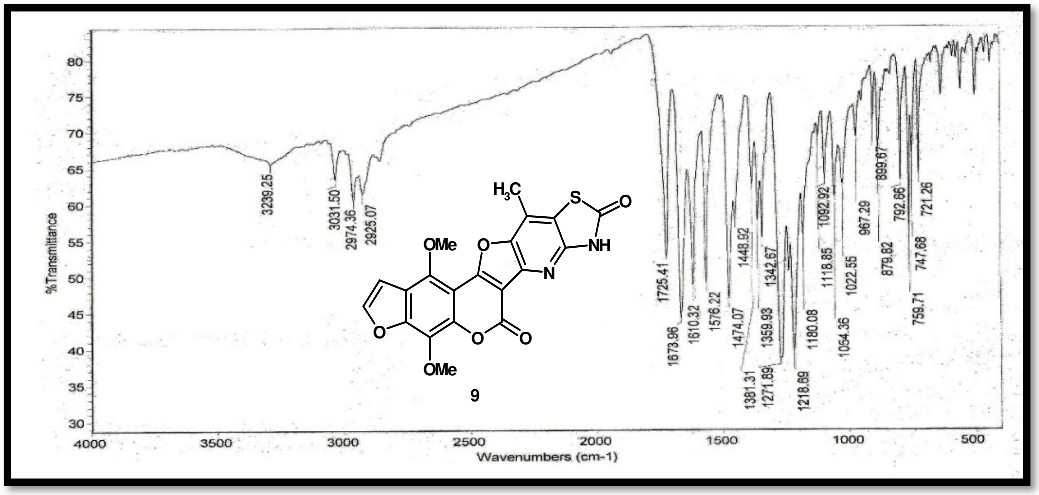

Figure S23: IR Spectrum of compound 9

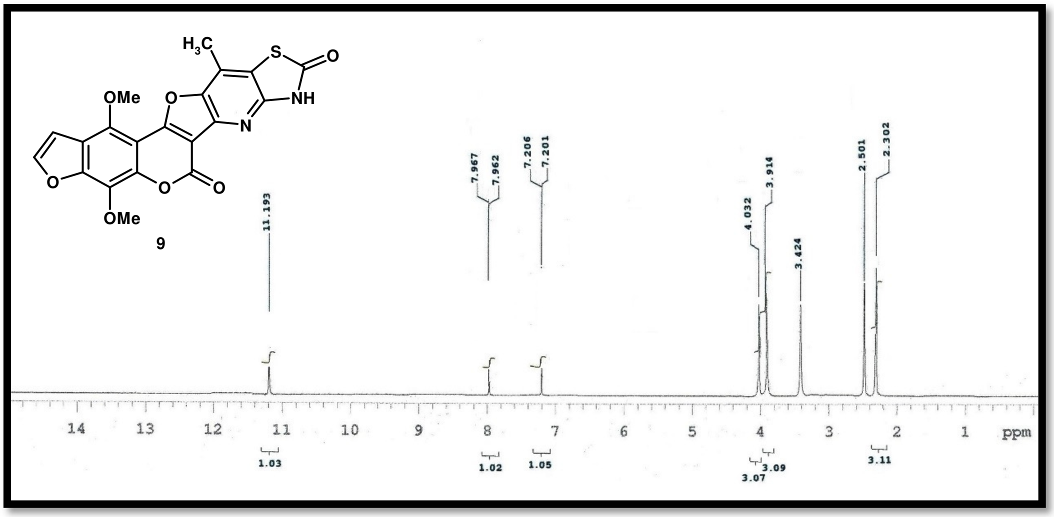

Figure S24 (DMSO-d_6, 400 MHz): ^{1}H NMR Spectrum of compound 9

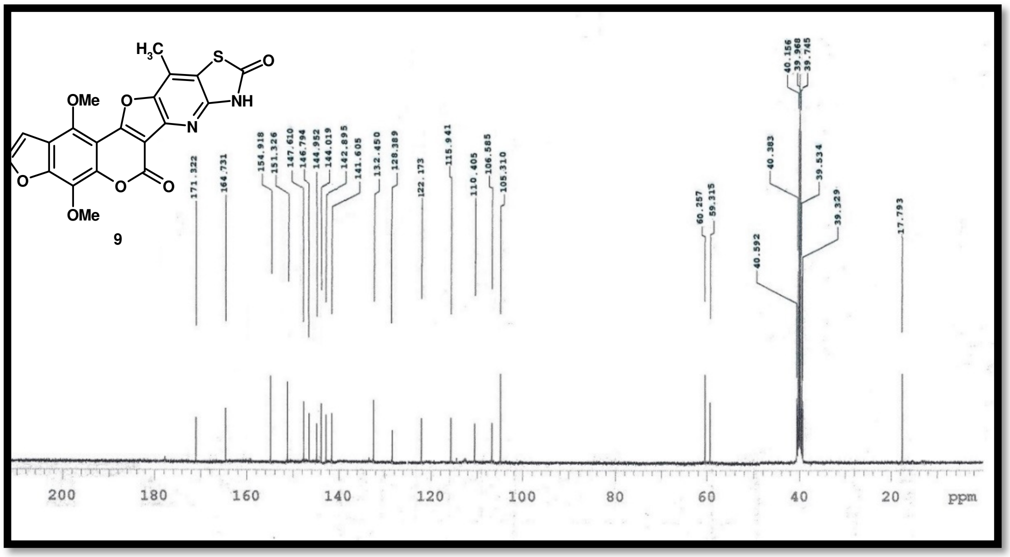

Figure S25 (DMSO-d_6, 100 MHz): ^{13}C NMR Spectrum of compound 9

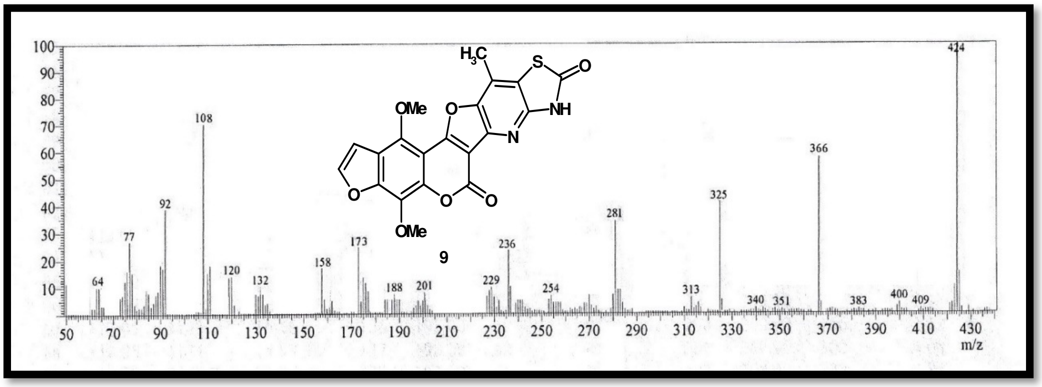

Figure S26: Mass Spectrum of compound 9

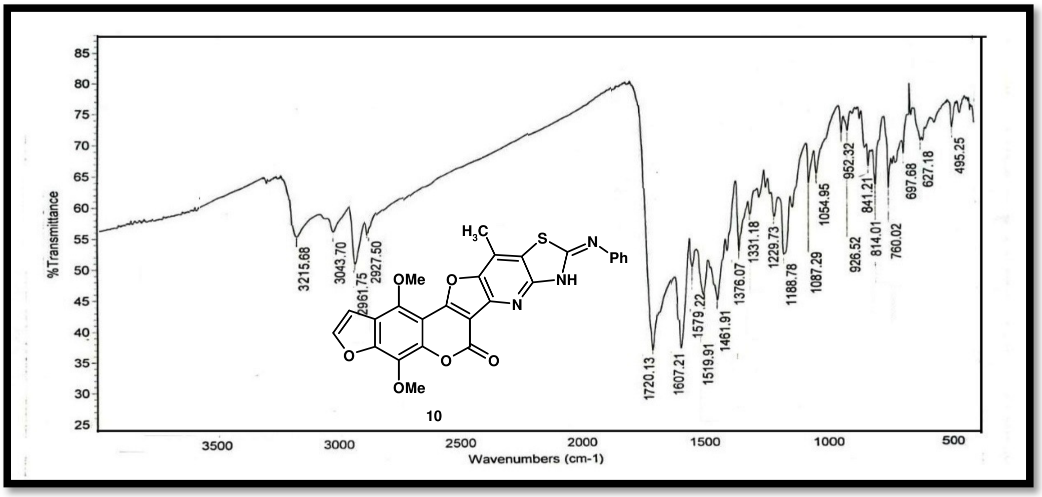

Figure S27: IR Spectrum of compound 10

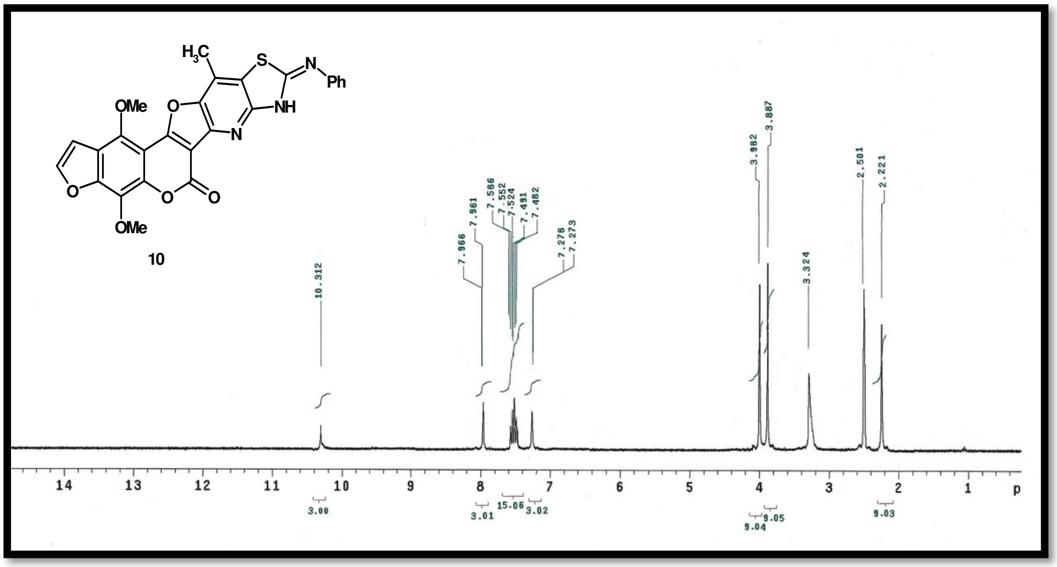

Figure S28 (DMSO-d_6, 400 MHz): ^{1}H NMR Spectrum of compound 10

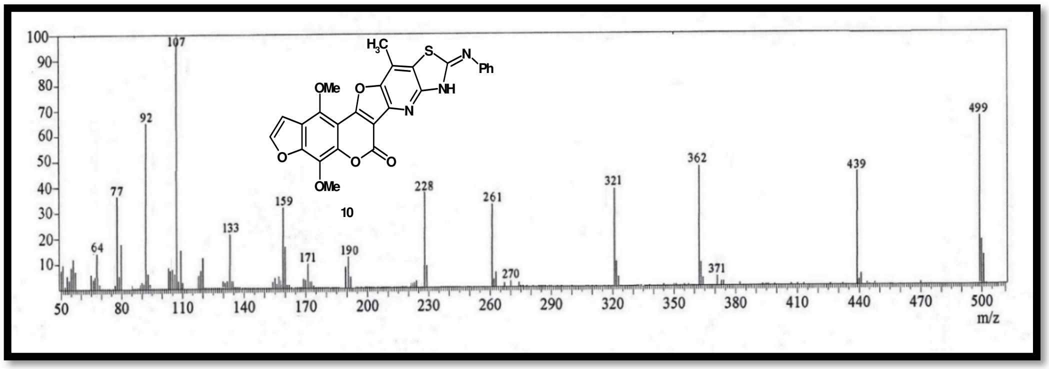

Figure S29: Mass Spectrum of compound 10

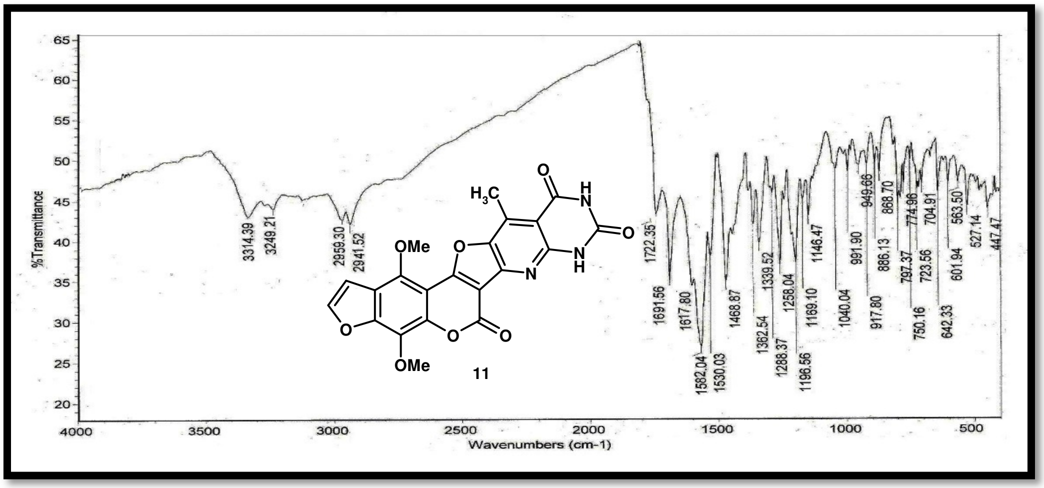

Figure S30: IR Spectrum of compound 11

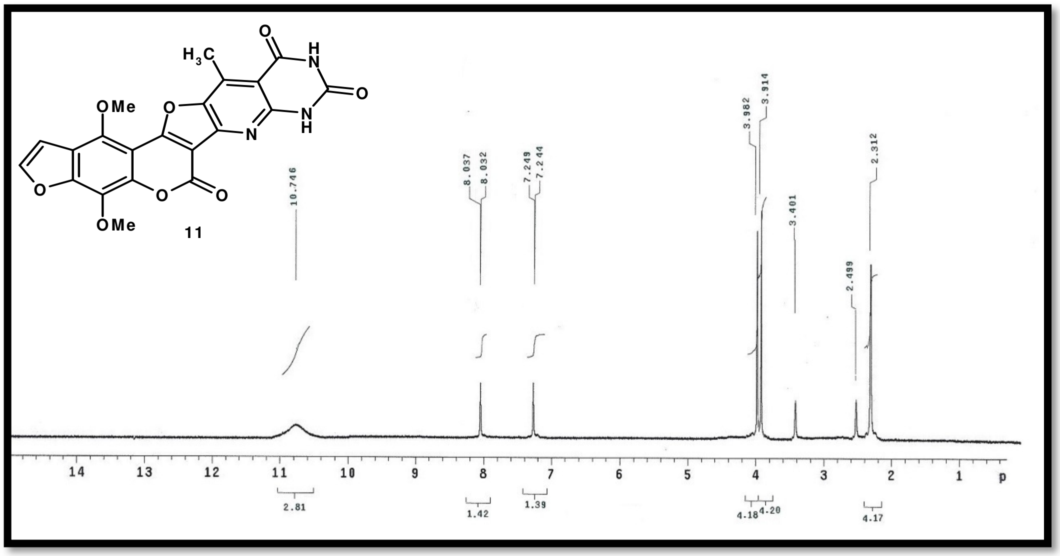

Figure S31 (DMSO-d_6, 400 MHz): ^{1}H NMR Spectrum of compound 11

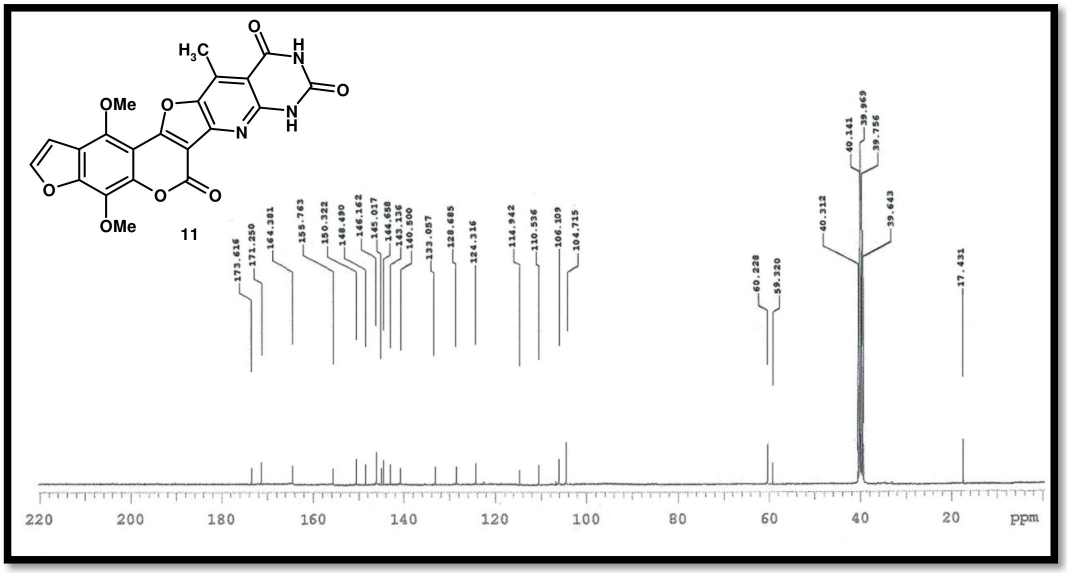

Figure S32 (DMSO-d_6, 100 MHz): ^{13}C NMR Spectrum of compound 11

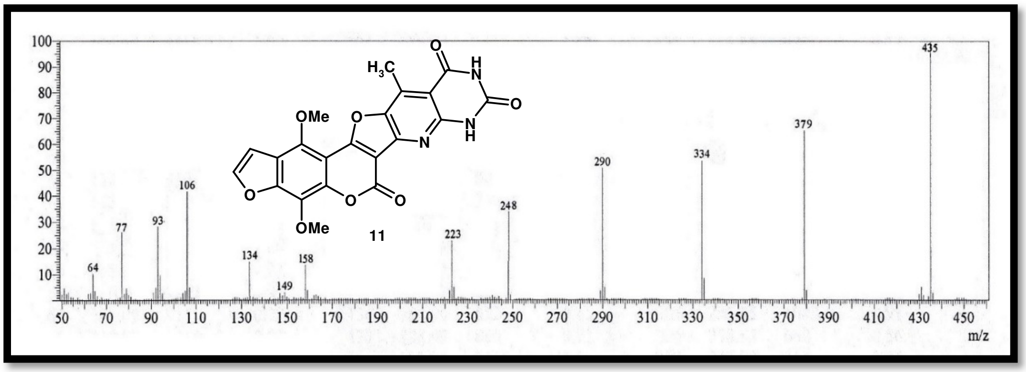

Figure S33: Mass Spectrum of compound 11

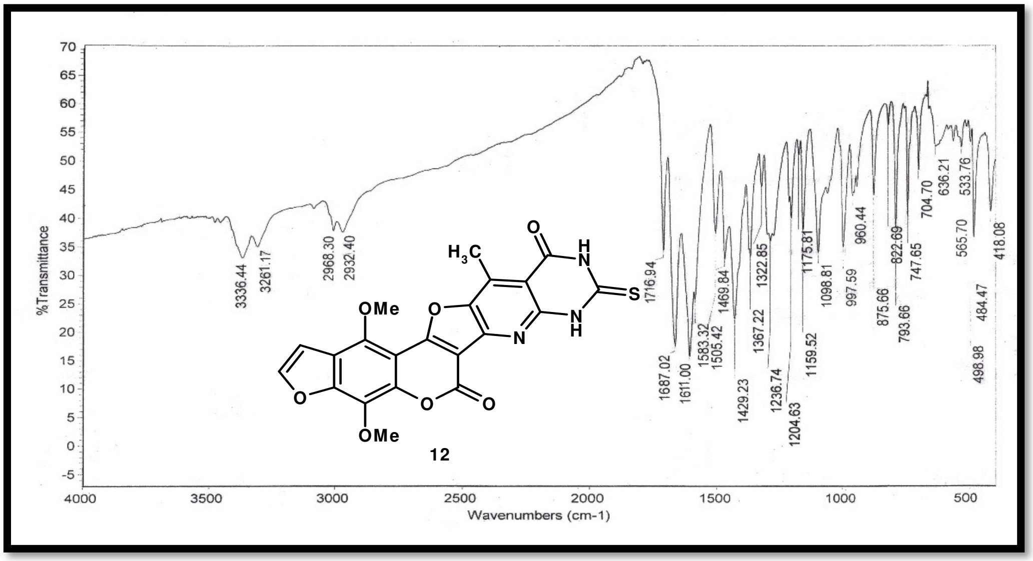

Figure S34: IR Spectrum of compound 12

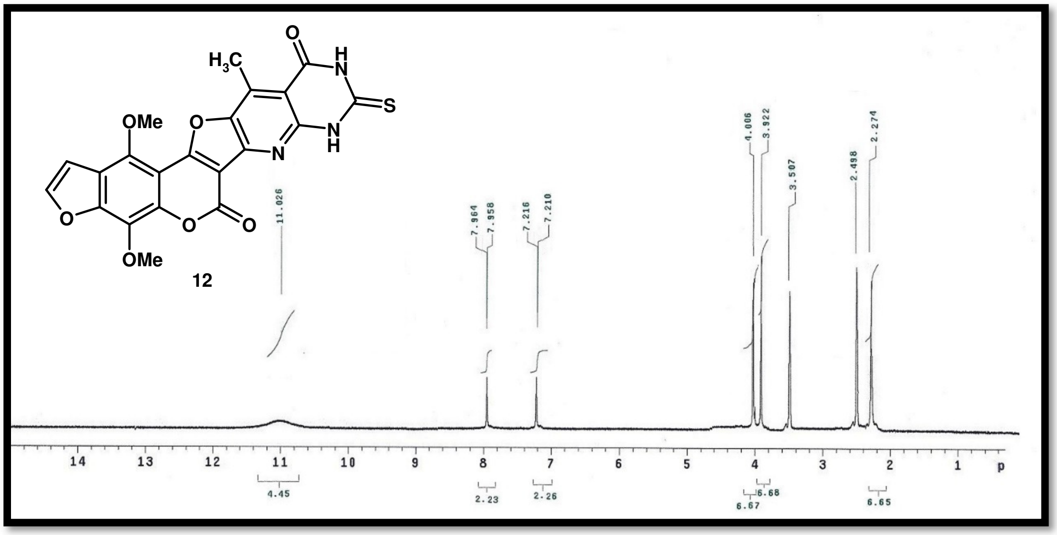

Figure S35 (DMSO-d_6, 400 MHz): ^{1}H NMR Spectrum of compound 12

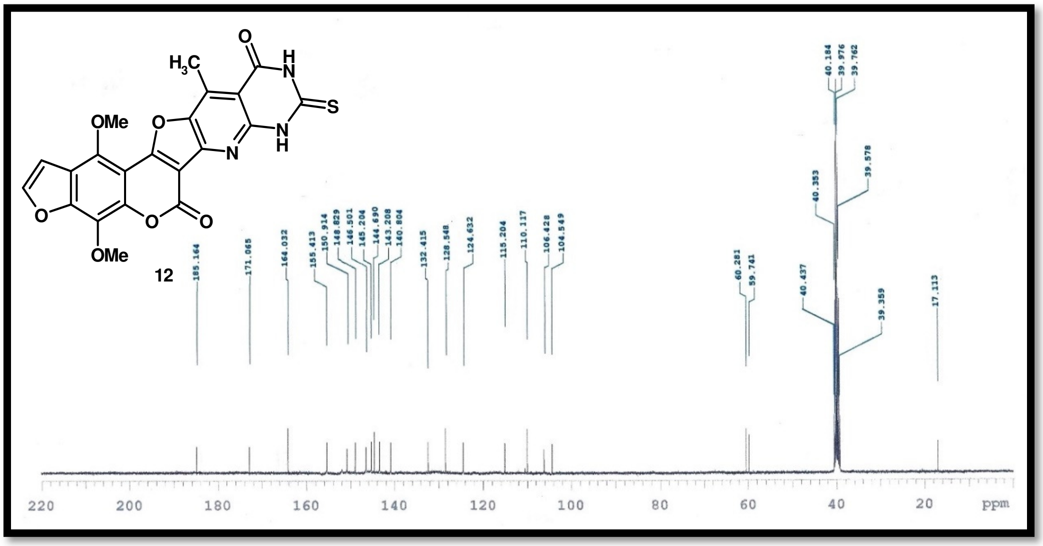

Figure S36 (DMSO-d_6, 100 MHz): ^{13}C NMR Spectrum of compound 12

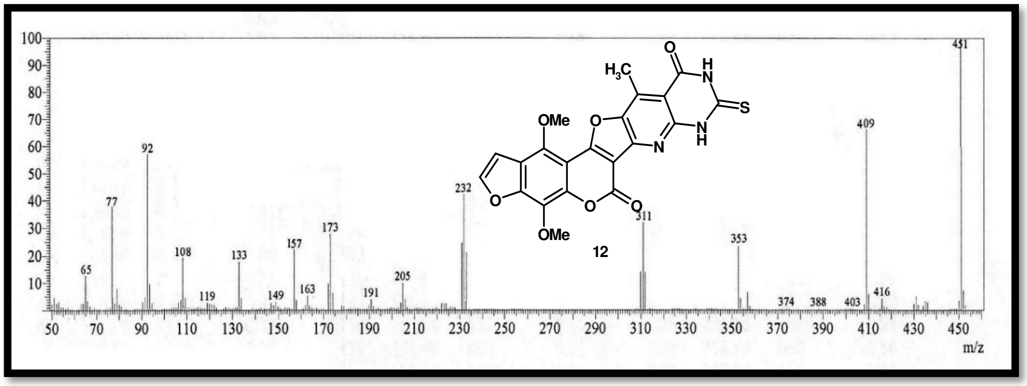

Figure S37: Mass Spectrum of compound 12

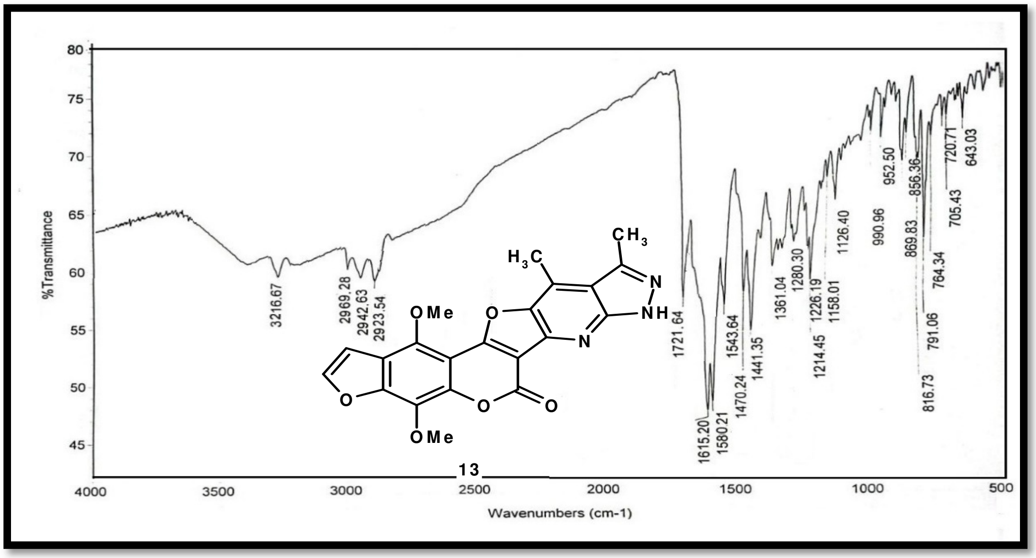

Figure S38: IR Spectrum of compound 13

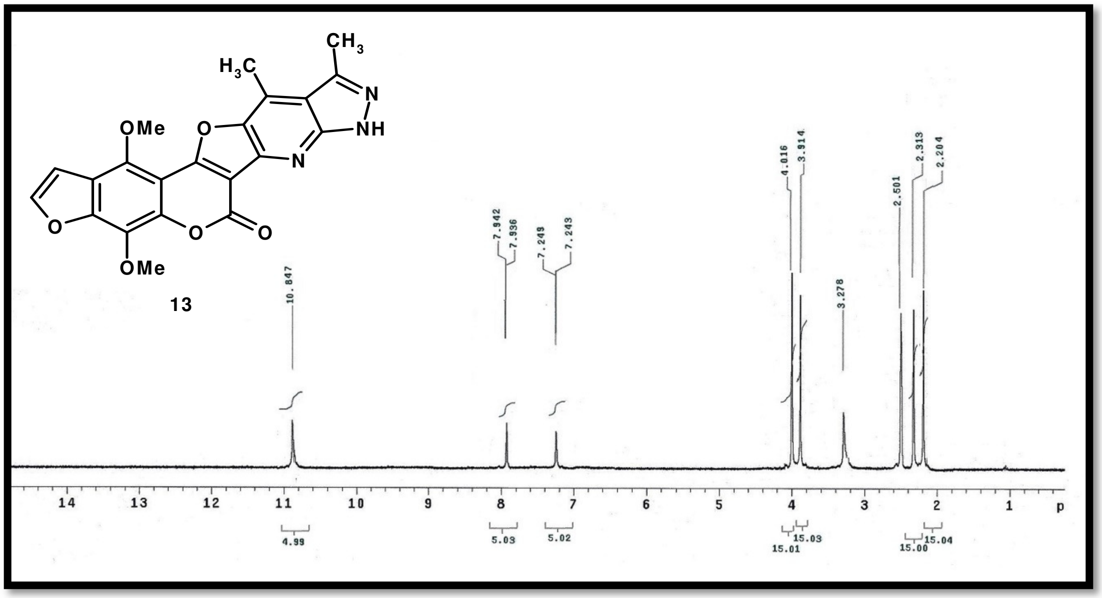

Figure S39 (DMSO-d_6, 400 MHz): ^{1}H NMR Spectrum of compound 13

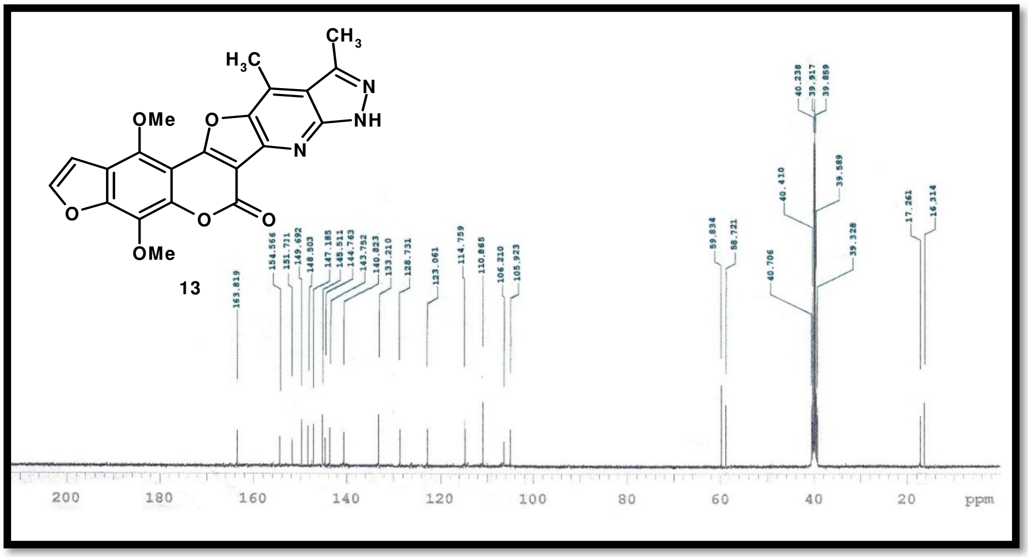

Figure S40 (DMSO-d_6, 100 MHz): ^{13}C NMR Spectrum of compound 13

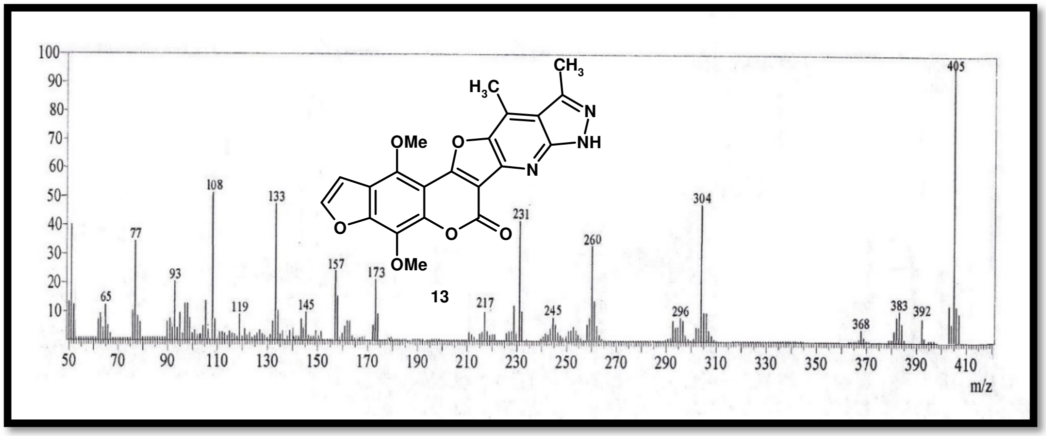

Figure S41: Mass Spectrum of compound 13

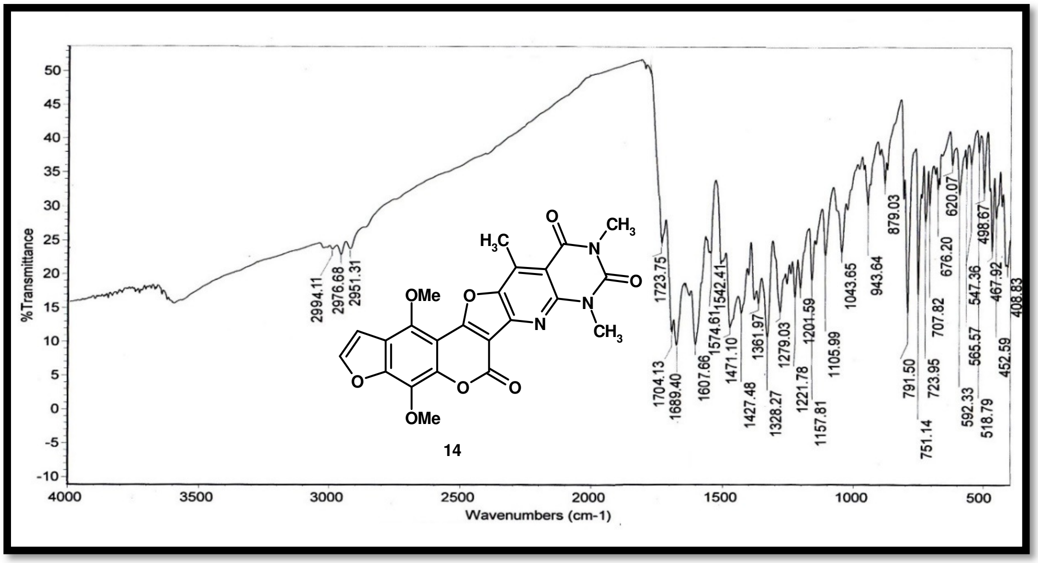

Figure S42: IR Spectrum of compound 14

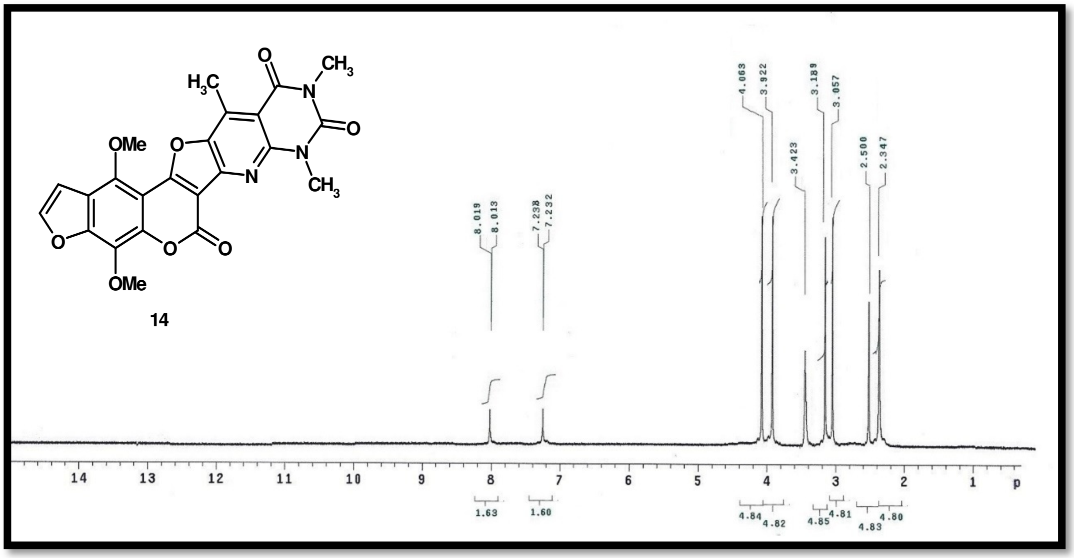

Figure S43 (DMSO-d_6, 400 MHz): ^{1}H NMR Spectrum of compound 14

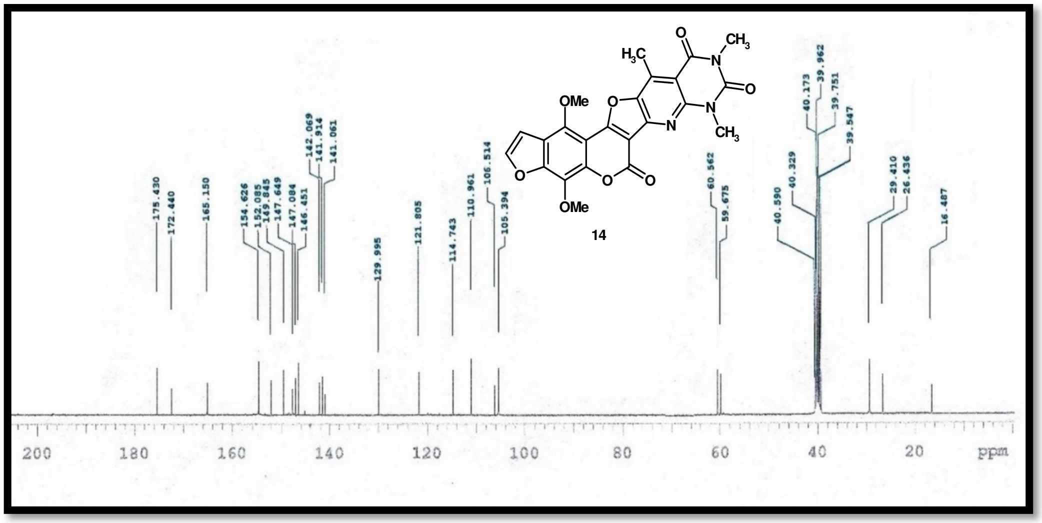

Figure S44 (DMSO-d_6, 100 MHz): ^{13}C NMR Spectrum of compound 14

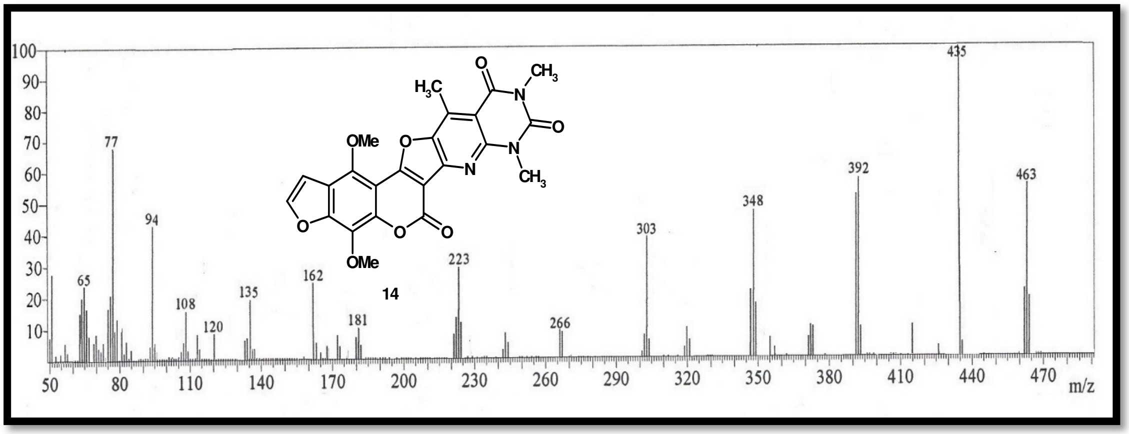

Figure S45: Mass Spectrum of compound 14
